# A mapped dataset of surface ocean acidification indicators in large marine ecosystems of the United States

**DOI:** 10.1038/s41597-024-03530-7

**Published:** 2024-07-02

**Authors:** Jonathan D. Sharp, Li-Qing Jiang, Brendan R. Carter, Paige D. Lavin, Hyelim Yoo, Scott L. Cross

**Affiliations:** 1 Cooperative Institute for Climate, Ocean, and Ecosystem Studies, University of Washington, Seattle, WA 98195 USA; 2 NOAA/OAR Pacific Marine Environmental Laboratory, Seattle, WA 98115 USA; 3 Cooperative Institute for Satellite Earth System Studies, Earth System Science Interdisciplinary Center, University of Maryland, College Park, MD 20740 USA; 4 NOAA/NESDIS National Centers for Environmental Information, Silver Spring, MD 20910 USA; 5 NOAA/NESDIS Center for Satellite Applications and Research, College Park, MD 20740 USA; 6 NOAA/NESDIS National Centers for Environmental Information, Charleston, SC 29412 USA

**Keywords:** Marine chemistry, Ocean sciences, Scientific data

## Abstract

Mapped monthly data products of surface ocean acidification indicators from 1998 to 2022 on a 0.25° by 0.25° spatial grid have been developed for eleven U.S. large marine ecosystems (LMEs). The data products were constructed using observations from the Surface Ocean CO_2_ Atlas, co-located surface ocean properties, and two types of machine learning algorithms: Gaussian mixture models to organize LMEs into clusters of similar environmental variability and random forest regressions (RFRs) that were trained and applied within each cluster to spatiotemporally interpolate the observational data. The data products, called RFR-LMEs, have been averaged into regional timeseries to summarize the status of ocean acidification in U.S. coastal waters, showing a domain-wide carbon dioxide partial pressure increase of 1.4 ± 0.4 μatm yr^−1^ and pH decrease of 0.0014 ± 0.0004 yr^−1^. RFR-LMEs have been evaluated via comparisons to discrete shipboard data, fixed timeseries, and other mapped surface ocean carbon chemistry data products. Regionally averaged timeseries of RFR-LME indicators are provided online through the NOAA National Marine Ecosystem Status web portal.

## Background & Summary

The accumulation of carbon dioxide (CO_2_) in the atmosphere as a result of human activities, and the uptake of ~25% of anthropogenic CO_2_ by the ocean^1,2^, has led to increasing acidity of ocean waters of about −0.016 pH units per decade on a global scale since the 1980s^3–6^. This ocean acidification (OA) signal is measurable at time series sites^7,8^, observed in mapped data products of CO_2_ partial pressure^6,9–12^, captured by decadal repeat hydrographic cruises^13,14^, and simulated by ocean models^15^ and coupled Earth system models^5,16,17^. Superimposed on steady increases in accumulated anthropogenic carbon (*C*_ant_) and decreases in ocean pH, however, are various modes of temporal (e.g., diurnal, seasonal, interannual) and spatial (e.g., latitudinal, nearshore–offshore) variability, which are particularly pronounced in coastal ecosystems. This variability in coastal OA brings unique impacts to marine organisms that reside in coastal zones and are vulnerable to corrosive waters^18^.

Large marine ecosystems (LMEs) are ocean regions that border coastlines and are characterized by distinct bathymetry, hydrography, productivity, and trophic structure^19^. LMEs encompass estuaries and river mouths, nearshore coastal zones, continental shelves, and the outer margins of ocean current systems. Typically, the offshore boundary of an LME extends to the continental shelf break or to the seaward edge of a current system. Due to their coastal proximity, LMEs tend to be natural hotspots of variability in carbon cycling and rapid exchange between carbon pools. For example, intense surface primary productivity in the coastal ocean is fueled by nutrients from river input, atmospheric deposition, and coastal upwelling^18^; sinking organic matter from surface production leads to intense respiration throughout the water column and at the seafloor^20^; and high rates of sedimentation are observed in LMEs from both biogenic and lithogenic inputs^21^.

Ongoing anthropogenic climate drivers coupled with the natural processes occurring in coastal ecosystems make it challenging to attribute modes of OA variability to the appropriate driving mechanisms. For example, anthropogenic eutrophication from freshwater runoff and atmospheric pollution can augment natural nutrient inputs, leading to even greater net primary production in coastal surface waters and greater respiration in subsurface waters. Whereas the direct effect of CO_2_ uptake by primary producers mitigates OA at the surface, highly respired subsurface waters can be laterally transported and upwelled onto the continental shelf, leading to enhanced OA in the surface waters there^18,22^. These and other OA-modulating processes differ across ecosystems^23^, but their impacts are frequently correlated with environmental driver variables such as sea surface height, temperature, salinity, and chlorophyll-a concentration. These correlations allow OA metrics to be reconstructed from measurements and data products that are available at high spatial and temporal resolution^11,24,25^.

The data product described here is based on direct observations, which are used to reconstruct a recent history of surface ocean OA indicators at monthly, 0.25° resolution in U.S. LMEs. Observations are from a publicly available, annually updated database of surface CO_2_ observations: the Surface Ocean CO_2_ Atlas (SOCAT)^26^. SOCAT is an international data synthesis effort that has facilitated the production of global surface CO_2_ flux maps^27^ that contribute data-constrained estimates of the ocean CO_2_ sink in the Global Carbon Budget^28^. We also rely on publicly available satellite-derived surface ocean properties and data reanalysis products to leverage the predictive power of environmental variables for upscaling SOCAT observations across U.S. LMEs. This kind of spatiotemporal upscaling has historically been accomplished using statistical interpolations^29,30^, multiple linear regressions^31^, and machine learning approaches^9,24,32,33^. We build upon the approach of Sharp *et al*.^24^ — who presented a monthly surface ocean CO_2_ partial pressure (*p*CO_2_) mapped product for the California Current System region called RFR-CCS — to train random forest regression (RFR) algorithms to predict surface CO_2_ fugacity (*f*CO_2_) from environmental variables that can be derived with spatial and temporal continuity across U.S. LMEs.

We advance the Sharp *et al*.^24^ approach by first clustering each LME into sub-regions with similar environmental variability using Gaussian mixture modelling. In addition to *f*CO_2_, we predict surface total alkalinity (and nutrients) from empirical property estimation algorithms that have been validated and published^34^. We use *f*CO_2_ and total alkalinity (*A*_T_) to compute eight additional OA indicators — partial pressure of CO_2_, total dissolved inorganic carbon, pH on the total scale, hydrogen ion amount content, carbonate ion amount content, saturation states for aragonite and calcite, and the Revelle factor — to produce monthly data products over 1998–2022 on a 0.25° × 0.25° resolution grid. We refer to these data products as RFR-LMEs^35^, which are freely available online and will be updated annually. Throughout this paper, we will use the term “mapped data products” to describe RFR-LMEs; “mapping” refers to the reconstruction of OA indicators on monthly, spatially continuous grids via the two-step approach of clustering on regional variability and applying trained RFR algorithms to gridded predictor variables.

This work was partially motivated by a partnership with the National Oceanic and Atmospheric Administration (NOAA) Ecosystem Indicators Working Group (EIWG), who manage the National Marine Ecosystem Status (NaMES) website (https://ecowatch.noaa.gov). The NaMES website was created to provide an at-a-glance overview of conditions in U.S. LMEs. These conditions are presented as indicators, which are quantitative and/or qualitative measures of key components of the ecosystem and span the following categories: climatological (e.g., El Niño Southern Oscillation index), physical–chemical (e.g., sea surface temperature), biological (e.g., chlorophyll-a concentration), and human dimensions (e.g., coastal county population). Indicator datasets are used by many NOAA stakeholders, such as fisheries managers, to monitor their ecosystems of interest and to assess the potential for future changes. Indicators included on the NaMES website must be theoretically sound, have demonstrable importance to the system, be relevant and understandable, show sensitivity to environmental variability or policy actions, and complement other indicators that are already served. This paper will describe the theoretical basis of RFR-LMEs and their relevance their respective ecosystems, to justify the use of RFR-LMEs as NaMES indicators of ocean acidification. The NaMES requirements also state that the data used to develop ecosystem indicators should be publicly available, quantitative, directly measurable, and updated on a regular basis; they stipulate that data should have adequate spatial coverage and that the time-series duration should be greater than 10 years and expected to continue for the foreseeable future. Because RFR-LMEs fit these requirements, we aggregate three mapped OA indicators from RFR-LMEs into monthly and annual regional averages of those indicators (and their uncertainties). Timeseries of the selected OA indicators (*p*CO_2_, pH on the total scale, and aragonite saturation state) are available on the NaMES website and, like the RFR-LME mapped data products, will be updated annually.

## Methods

**Fig. 1:**
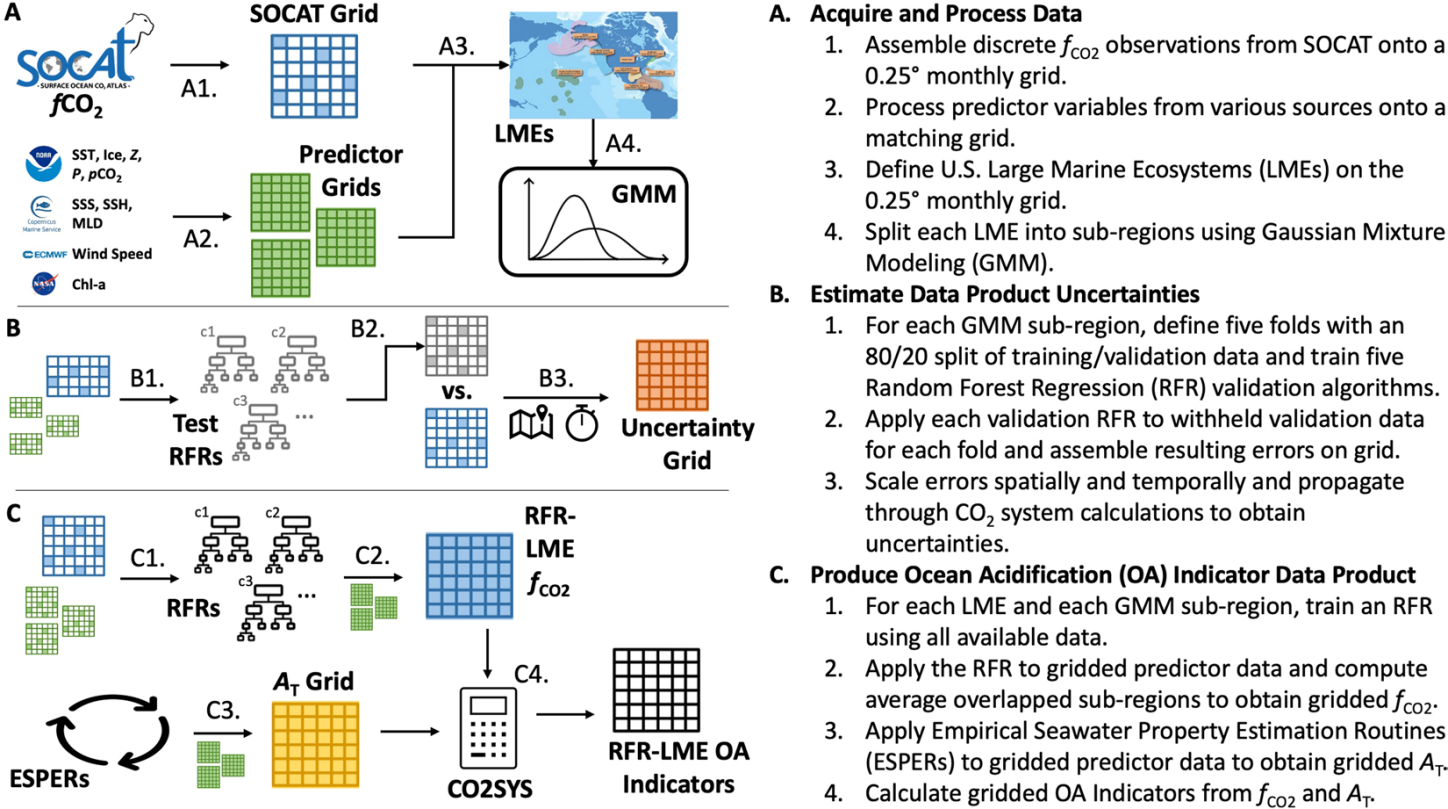
Schematic of the procedure used to construct the ocean acidification indicator data products described by this study. Steps within each section on the right (**A**, **B**, and **C**) are labelled in the schematic on the left.

An overview of the methodological procedure to create RFR-LMEs is provided in Fig. 1. First, data were obtained from a variety of sources and bin-averaged or interpolated onto a consistent grid. Then, within each LME, a two-step cluster–regression strategy was employed. In the first step, spatial clusters were created using Gaussian mixture models (GMMs) based on variability in environmental predictors. In the second step, random forest regression (RFR) algorithms were trained for each cluster using *f*CO_2(SOCAT)_ as the target variable and co-located environmental variables as predictors. These algorithms were then applied to gridded (0.25° × 0.25°) monthly environmental predictor fields to create monthly RFR-LME mapped data products of sea surface CO_2_ fugacity (*f*CO_2(RFR-LME)_). Applying GMMs on surface data to first divide each LME into subregions reduces the burden on the RFRs to represent many different regimes of dynamic variability at once. Therefore, the RFR algorithms are able to reconstruct sea surface *f*CO_2_ more accurately than if all data points from the entire LME were included in the algorithm training^36^. To create RFR-LMEs for the other indicators, sea surface total alkalinity and nutrient values were estimated, and carbonate system calculations were performed. Uncertainties were propagated through these calculations to obtain uncertainty estimates for each RFR-LME. Finally, RFR-LMEs were evaluated against independent datasets.

### Data sources

**Fig. 2:**
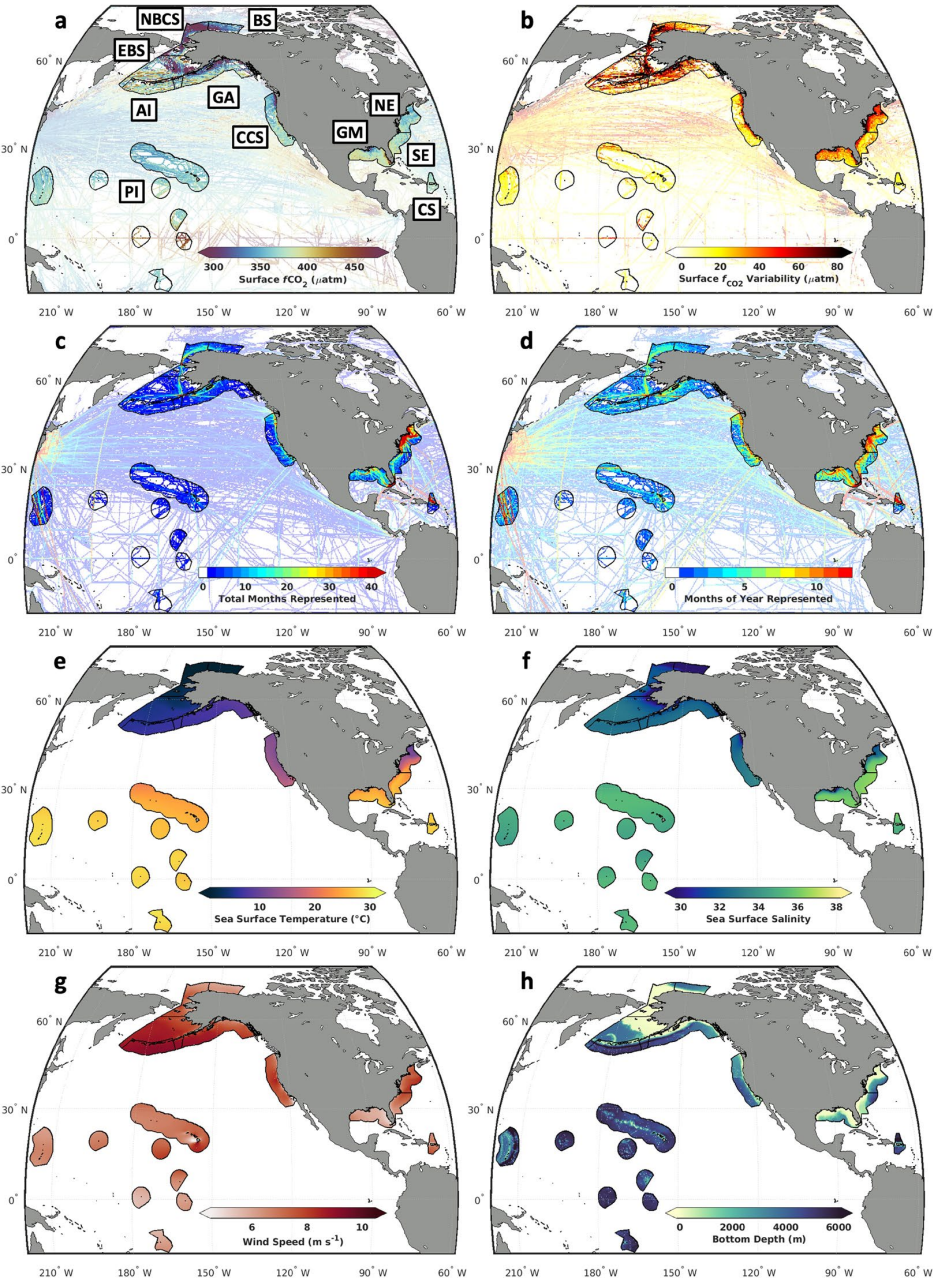
Observations used to develop ocean acidification indicator data products in the eleven U.S. large marine ecosystems (LMEs) considered in this study. LME boundaries are displayed with (**a**) platform-weighted *f*CO_2_ from SOCATv2023, averaged over 1998–2022 in 0.25 degrees latitude by 0.25 degrees longitude grid cells; (**b**) platform-weighted *f*CO_2_ variability from SOCATv2023, calculated as the standard deviation over time in 0.25 degrees latitude by 0.25 degrees longitude grid cells; (**c**) the total number months over the timeseries with at least one observation in each grid cell; (**d**) months of the seasonal cycle with at least one observation in each grid cell; (**e**) sea surface temperature from OISSTv2^38^; (**f**) sea surface salinity from the CMEMS GLORYS reanalysis product^39^; (**g**) wind speed from the ECMWF ERA5 reanalysis product^43^; and (**h**) bathymetry from the ETOPO 2022 Global Relief Model^44^. LME name abbreviations shown in panel (**a**) are provided in Table 1.

Surface ocean *f*CO_2_ observations were downloaded from the Surface Ocean CO_2_ Atlas Version 2023 (SOCATv2023; 10.25921/r7xa-bt92)^37^ in a large quadrangle surrounding North America and U.S. Pacific Islands with the following coordinates: 18°S to 82°N, 140°E to 58°W (Fig. 2). These observations were filtered by year (1998–2022), dataset flag (A, B, C, or D), and quality flag (q.f. = 2, good data), and binned into 0.25 degrees latitude by 0.25 degrees longitude monthly grid cells using platform-weighted averages. A spatial resolution of 0.25° × 0.25° was chosen largely for coherence with the majority of available predictor datasets. Platform-weighted averages mean that, within each latitude by longitude by month bin, a platform-specific (e.g., ship-only, mooring-only) average was first calculated, then an average was taken of those averages (if more than one platform was represented within the cell). This was done to mitigate unwanted biases toward high-resolution measurement systems. For validation exercises, this binning process was also repeated with only moored buoy observations and with a dataset that excluded moored buoy observations.

Binned observations were grouped into eleven LMEs defined according to the United States Exclusive Economic Zone (EEZ), in accordance with the practice of the NOAA EIWG (Table 1; Fig. 2). Platform-weighted *f*CO_2_ from SOCATv2023 observations (*f*CO_2(SOCAT)_) in each of these grid cells over time shows large-scale patterns of spatial variability (Fig. 2a) — such as relatively high *f*CO_2(SOCAT)_ at the equator and relatively low *f*CO_2(SOCAT)_ surrounding Alaska, compared to the region as a whole — and temporal variability (Fig. 2b) — such as relatively high standard deviation in *f*CO_2(SOCAT)_ observations surrounding Alaska and near the coastlines of the continental U.S. compared to the relatively low standard deviation in these observations around the Pacific Islands, again compared to the region as a whole. The distribution of the total number of months sampled within each 0.25° × 0.25° grid cell and the number of months of the year sampled at least once across the full dataset (1998–2022) within each grid cell reveal consistent patterns (Fig. 2c,d). The Northeast U.S. has especially high observational coverage (9.5% of all 0.25° × 0.25° monthly grid cells covered and 63.6% of seasonal seasonally binned 0.25° × 0.25° grid cells covered); the Southeast U.S. (4.0% total, 50.0% seasonal), Gulf of Mexico (5.2% total, 53.1% seasonal), Caribbean Sea (6.6% total, 40.2% seasonal), and California Current System (3.0% total, 42.1% seasonal) have moderately high observational coverage (Table 1). Observational coverage generally decreases farther offshore.

**Table 1:** Summary information for the eleven U.S. large marine ecosystems (LMEs) considered in this study.

Large Marine Ecosystem (LME)	Abbrev.	Area (10^6^ km^2^)	Total Data Coverage (%)	Seasonal Data Coverage (%)	*N*
**California Current**	CCS	0.81	3.0	42.1	3
**Gulf of Alaska**	GA	1.00	1.7	25.5	5
**Aleutian Islands**	AI	0.67	0.8	18.4	4
**East Bering Sea**	EBS	1.40	1.1	18.7	4
**Beaufort Sea**	BS	0.24	1.4	17.6	5
**Chukchi and Northern Bering Seas**	NBCS	0.46	2.6	27.3	6
**Northeast U.S**.	NE	0.46	9.5	63.6	4
**Southeast U.S**.	SE	0.42	4.0	49.9	3
**Gulf of Mexico**	GM	0.70	5.2	53.1	5
**U.S. Caribbean**	CS	0.21	6.6	40.2	3
**Pacific Islands**	PI	5.79	1.1	15.4	4

Areas are calculated as the sum area of all 0.25° × 0.25° grid cells that fall within the limits of each LME, taking into account the percentage of each grid cell covered by ocean determined via the ETOPOv2022 Global Relief Model. Data coverage is calculated as the percentage of monthly grid cells occupied by an observation over the entire time series (Total) and binned over a seasonal cycle (Seasonal). The number of Gaussian Mixture Model (GMM) clusters (*N*) for each LME is determined by the GMM optimization procedure described in the *Spatial clustering* section.

Next, gridded fields of satellite, reanalysis, and *in situ* observational products were downloaded from the sources detailed in Table 2. When applicable, these fields were re-gridded using standard interpolation functions to match the resolution and/or central grid cell positions of the binned *f*CO_2(SOCAT)_ observations. In many cases, multiple datasets could be chosen, but preference were given to those that were provided at 0.25° resolution and that covered the relevant time and space. Rigorous comparison between different input datasets is planned for future development of RFR-LMEs as they are prepared for dynamic, operational production. Sea surface temperature (SST; Fig. 2e) and ice concentration were obtained from the NOAA Optimum Interpolation Sea Surface Temperature version 2 (OISSTv2) product at daily, 0.25° × 0.25° resolution^38^; values were averaged to monthly resolution. Sea surface salinity (SSS; Fig. 2f) and mixed layer depth (MLD) were obtained from the Copernicus Marine Environment Monitoring Service (CMEMS) Global Ocean Ensemble Physics Reanalysis (GLORYS) product at monthly, 0.25° resolution^39^. Sea surface height (SSH) was obtained from the CMEMS satellite gridded product, which is produced at monthly, 0.25° resolution by optimal interpolation of along-track measurements from available altimeter missions^40^. Sea surface chlorophyll (CHL) was obtained from the National Aeronautics and Space Administration (NASA) Ocean Colour Level-3 Mapped Chlorophyll Data product at monthly, 1/12° resolution and re-gridded to 0.25° resolution^41,42^. One-dimensional, linear interpolation was used within each grid cell to fill gaps in the chlorophyll dataset. Wind speed (Fig. 2g) was obtained from the fifth generation European Centre for Medium-Range Weather Forecasts (ECMWF) reanalysis for the global climate and weather (ERA5) at monthly, 0.25° resolution^43^. Bathymetry (*Z*, Fig. 2h) was obtained from the ETOPOv2022 Global Relief Model at 1/60° resolution and re-gridded to 0.25° resolution^44^. Sea level pressure (SLP) was obtained from the National Centers for Environmental Prediction/Department of Energy (NCEP/DOE) Reanalysis II model at monthly, 2.5° resolution and interpolated to 0.25° resolution^45^. Atmospheric *p*CO_2_ was obtained from the NOAA Marine Boundary Layer (MBL) product at weekly resolution and varying latitudinal resolution and was re-gridded to monthly, 0.25° resolution^46^. Binned observations of *f*CO_2(SOCAT)_ were co-located in both time and space with the gridded predictors in preparation for algorithm training.

**Table 2:** Sources of gridded fields of satellite, reanalysis, and *in situ* observational products used to create RFR-LME maps.

Variable	Dataset	DOI	Original Resolution (Lat. × Lon. × Time)
Temperature*^,#^	NOAA OISSTv2^38^	10.1175/JCLI-D-20-0166.1	0.25° × 0.25° × daily
**Ice Concentration**	NOAA OISSTv2^38^	10.1175/JCLI-D-20-0166.1	0.25° × 0.25° × daily
**Salinity**	CMEMS GLORYS^39^	10.48670/moi-00024	0.25° × 0.25° × monthly
**Mixed Layer Depth**	CMEMS GLORYS^39^	10.48670/moi-00024	0.25° × 0.25° × monthly
**Sea Surface Height**^**+**^	CMEMS L4^40^	10.48670/moi-00148	0.25° × 0.25° × monthly
Chlorophyll*^,+^	NASA L3^41,42^	10.5067/AQUA/MODIS/L3M/CHL/2022; 10.5067/ORBVIEW-2/SEAWIFS/L3M/CHL/2022	9 km × 9 km × monthly
**Wind Speed**^**#**^	ECMWF ERA5^43^	10.24381/cds.f17050d7	0.25° × 0.25° × monthly
**Bathymetry**	NOAA ETOPO 2022^44^	10.25921/fd45-gt74	1/30° × 1/30° × N/A
Sea Level Pressure*^,#^	NCEP-DOE AMIP-II^45^	10.1175/BAMS-83-11-1631	2.5° × 2.5° × monthly
**Atmospheric CO**_**2**_	NOAA MBL^46^	10.15138/wkgj-f215	*sin*(Lat.) = 0.5 × N/A × weekly

The digital object identifiers and original three-dimensional resolutions of each product are provided.

*Used for GMM clustering in most LMEs.

^#^Used for GMM clustering in BS and NBCS.

^+^Not used for RFR training in the BS or NBCS.

As one of several validation exercises, the *p*CO_2_ reconstructions from our method were compared to other mapped *p*CO_2_ data products downloaded from SeaFlux (v2021.04)^47^, which is an ensemble of six surface *p*CO_2_ products that enables users to calculate air–sea CO_2_ flux consistently across the global ocean^27^. SeaFlux harmonizes six data-based *p*CO_2_ products: CMEMS-FFNN^9,48^, MPI-SOMFFN^33,49^, and NIES-FNN^50^, which are each constructed using neural networks; JENA-MLS^30^, which is constructed based on a mixed layer scheme; JMA-MLR^10^, which is constructed using multiple linear regressions; and CSIR-ML6^36^, which is constructed using an ensemble of multiple machine-learning techniques. In an additional exercise, RFR-LME mapped data products were evaluated through comparisons to co-located, independently calculated OA indicators from research cruises included in the Global Ocean Data Analysis Project database (GLODAPv2.2022)^51^ and the Coastal Ocean Data Analysis Project – North America database (CODAP-NA)^52^. Measurements of SST, SSS, *A*_T_, *C*_T_, and nutrients were obtained from the GLODAP and CODAP-NA databases, then filtered to retain only observations with good quality flags for each of those variables that were collected at a depth of 10 meters or less.

### Spatial clustering

Three clustering methods were tested: self-organizing mapping — a neural-network-based method of producing a low-dimensional representation of a set of input data — *k*-means clustering — an iterative method that optimizes a defined number of centroids by minimizing the in-cluster distances from the centroid for a multidimensional dataset — and Gaussian mixture modelling (GMM)^53^ — a method of clustering that assumes a multidimensional dataset is represented by a mixture of several Gaussian distributions with different properties. Of these methods, preliminary testing suggested GMM provided the best results in terms of *k*-fold cross-validated root-mean-square error (RMSE) in *f*CO_2_ (described in the following section) after RFRs were fit for each cluster. In addition, GMM clustering affords the benefit of providing probabilities that each spatiotemporal grid cell belongs within a given cluster instead of simply providing the cluster assignment to each grid cell as done in the other two clustering methods. These probabilities are used in our method to mitigate discontinuities at boundaries between clusters.

Variability (defined as the standard deviation within a spatial grid cell over time) in SLP, SST, and CHL were used as feature sets to form clusters in most LMEs; CHL was replaced with wind speed in two LMEs (BS and NBCS) due to insufficient CHL observations at high latitudes. The decisions to cluster based on variability over time instead of monthly values and to use the specified sets of variables were based on initial testing and optimization in terms of *k*-fold cross-validated RMSE in *f*CO_2_ (not shown). Future development of RFR-LMEs may continue to explore alternative clustering strategies.

**Fig. 3:**
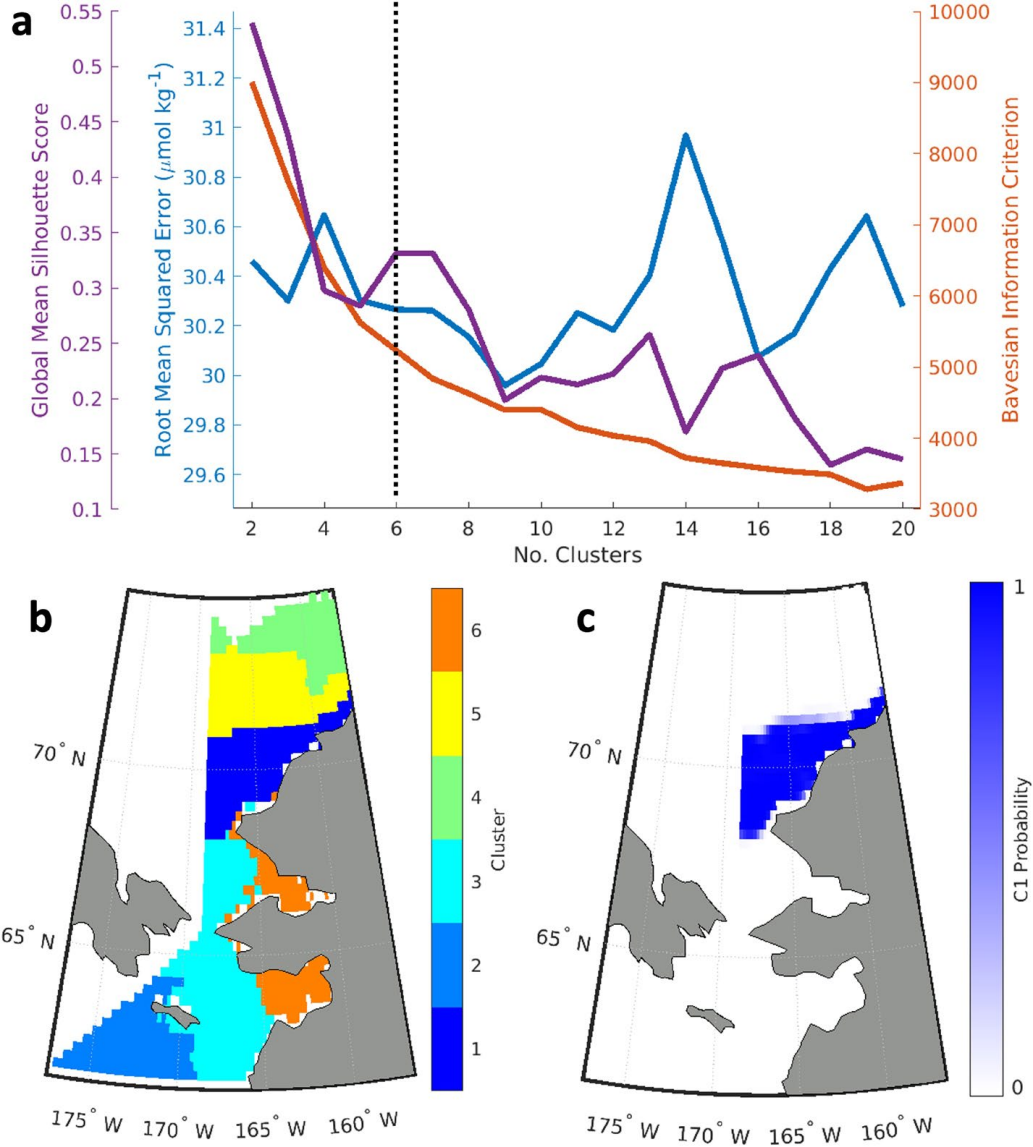
Example for Gaussian Mixture Model (GMM) optimization in the North Bering Chukchi Seas (NBCS). (**a**) Parameters used for GMM evaluation in the NBCS, plotted against the number of GMM clusters (*N*). The goal of the optimization procedure was to minimize the root mean squared error, maximize the global mean silhouette score, and identify the *N* at which the Bayesian information criterion was no longer sharply decreasing. *N* = 6 was ultimately selected for the NBCS region. (**b**) Distribution of GMM clusters for the NBCS and (**c**) the probability for grid cells belonging to cluster 1 (C1).

GMM models with full, unshared covariance matrices were created using the MATLAB “fitgmdist” function. Full covariance matrices were used for GMM based on the *a priori* assumption that some of the predictor variables were correlated due to the nature of oceanographic environmental variables. Covariance matrices for GMM were unshared based on the *a priori* assumption that each spatial cluster had its own, different covariance matrix. The number of components (i.e., clusters; *N* in Table 1) was optimized, primarily by minimizing the *k*-fold cross-validated RMSE in *f*CO_2_, but also taking into account the Bayesian information criterion — a measure of model fit that includes a penalty for the number of clusters — and silhouette score — a measure of the accuracy of the clustering technique that is calculated by comparing each point’s similarity to the other points in its assigned cluster to how dissimilar it is to the points in the next nearest cluster (Fig. 3).

### Machine learning regressions

Once the numbers of spatial clusters were determined for each LME, random forest regressions (RFRs)^54^ were trained for each cluster within each LME using binned *f*CO_2(SOCAT)_ as a target variable and each of the co-located gridded variables listed in Table 2 along with longitude (degrees east with a 0° to 360° convention), latitude, distance from the coast, month of the year (sine- and cosine-transformed to maintain cyclicity throughout the year and predictability within each month), and year as predictors. These variables were found to be useful predictors of *f*CO_2_ by Sharp *et al*.^24^.

RFRs are a collection of regression “trees”, each of which is trained with a bootstrapped subset of the dataset. Each tree aims to generate a representation of the relationship between the predictor variables and the target variable for its bootstrapped subset of the data. This is done by splitting the data into a series of “branches” based on the predictors. At each branch point, only a random subset of the predictor variables is made available to the algorithm. The algorithm then optimally selects a predictor dataset and a specific value from that dataset on which to split the dataset into two additional branches/groups with the lowest possible within-group *f*CO_2(SOCAT)_ variance. This continues until the branches become “leaves”, which means they are no longer split, either due to reaching a defined minimum leaf size or a certain criterion (e.g., variance of the remaining *f*CO_2(SOCAT)_ observations). The use of an ensemble of regression trees constitutes the “forest” aspect of an RFR. The “randomness” aspect of the forest is due to the fact that each tree is constructed with different subsets of the full dataset and that different subsets of the predictors are available at each branch point, making it possible for each tree to provide a slightly different empirical regression for the dataset. New predictor data can be passed through each tree in the ensemble of a trained RFR, and an average of the values output from each tree is the *f*CO_2_ prediction (*f*CO_2(RFR-LME)_).

For each cluster, all grid cells with a GMM probability of greater than 10% for that cluster were used to train an RFR using the MATLAB “TreeBagger” function. This means that many grid cells on the geographic boundary between one or more clusters may then have been used to train multiple RFRs. The number of trees used for each RFR was set to 1000, which was confirmed to be sufficient through visual inspection of the out-of-bag RMSE with respect to the number of trees (not shown). The minimum leaf size was set to three based on *k*-fold cross-validation testing, and the number of predictors used for each decision split was set to 6 (equal to the total number of predictors divided by three and rounded up to the nearest whole number).

To create an RFR-LME map of *f*CO_2_ for each LME, all the gridded predictor variables (0.25° × 0.25°, monthly) within the LME were run through each cluster-specific RFR. This produced *N f*CO_2(RFR-LME)_ maps for each LME, where *N* is equal to the number of clusters. These maps were then merged as weighted average *f*CO_2(RFR-LME)_ maps using the GMM probabilities as weights, which helped to smooth out discontinuities between clusters. Lastly, RFR-LME maps of *f*CO_2_ were converted to maps of *p*CO_2_ (*p*CO_2(RFR-LME)_) using SST and SLP^55^.

Cross-validation was used to evaluate the skill of the *f*CO_2(RFR-LME)_ estimates in each cluster and overall in each LME. This *k*-fold cross-validation was performed by sequentially withholding subsets of 20% of data, training versions of RFR algorithms with the remaining 80% of data, then, for each data point in the validation dataset, comparing the *f*CO_2_ obtained using the *k*-fold cross-validation algorithms (*f*CO_2(RFR-LME-kFold)_) to the observed *f*CO_2(SOCAT)_ value. This procedure was repeated five times for each LME so all data points were included in the validation data once, producing Δ*f*CO_2_ values for each data point.

### Alkalinity and nutrient estimation

Sea surface total alkalinity (*A*_T_), phosphate (PO_4_), and silicate (Si(OH)_4_) were estimated from gridded monthly fields of SSS and SST using Empirical Seawater Property Estimation Routines (ESPERs)^34^. ESPERs consist of both locally interpolated multiple linear regressions (ESPER-LIR) and feed-forward neural networks (ESPER-NN) trained to estimate seawater properties from a given set of input properties. Though ESPERs are global in nature, the regionally tuned ESPER-LIR coefficients and spatial coordinate predictors in ESPER-NNs mean that ESPERs function similarly to regional property estimation algorithms. ESPERs also provide the benefit of estimating uncertainty corresponding to each predicted value, allowing for the propagation of those uncertainties through downstream computations. The ESPER-Mixed routine (an average of both the ESPER-LIR and ESPER-NN approaches) was used for this study, due to assessment statistics that have indicated a lower global RMSE for the ESPER-Mixed approach (e.g., a global average RMSE of 3.7 μmol kg^−1^ for *A*_T_) compared to ESPER-LIR (4.0 μmol kg^−1^) and ESPER-NN (4.1 μmol kg^−1^) when producing property estimates from SSS and SST^34^.

### Carbonate system calculations

CO_2_ system calculations were performed using CO2SYSv3 for MATLAB^56^ to determine additional ocean acidification (OA) indicators: dissolved inorganic carbon (*C*_T(RFR-LME)_), pH on the total scale (pH_T(RFR-LME)_), total hydrogen ion amount content ([H^+^]_T(RFR-LME)_), total carbonate ion amount content ([CO_3_^2−^]_T(RFR-LME)_), saturation states for aragonite (Ω_ar(RFR-LME)_) and calcite (Ω_ca(RFR-LME)_), and Revelle factor (RF_(RFR-LME)_). These calculations were performed using well established thermodynamic equations describing the chemistry of carbon dioxide in seawater^57,58^. Input parameters to these equations were *f*CO_2(RFR-LME)_, along with ESPER-estimated *A*_T_ (*A*_T(ESPER)_), phosphate (PO_4(ESPER)_), and silicate (Si(OH)_4(ESPER)_). Carbonic acid dissociation constants from Lueker *et al*.^59^, the boric acid dissociation constant from Dickson^60^, the total boron to salinity ratio from Lee *et al*.^61^, the dissociation constant of water from Dickson^62^, and the hydrofluoric acid dissociation constant from Perez and Fraga^63^ were used in CO_2_ system calculations. Uncertainties were propagated through these calculations (see following section).

### Uncertainty estimation

Uncertainties in RFR-LME maps of *f*CO_2_ were evaluated based on the previously described *k*-fold cross-validation approach. First, spatially gridded absolute values of Δ*f*CO_2_ from *k*-fold cross-validation were low-pass filtered (using 0.5° × 0.5° windows) two times in each LME to begin to fill nearby grid cells with uncertainty values. Then, nearest-neighbor interpolation was used to fill any remaining empty grid cells with data-based, spatially scaled uncertainty values (${{\rm E}}_{{fCO}2(s)}$). This approach only assesses the strength of the fit for available. It is therefore prudent to assign greater uncertainties for periods and regions where training data are less abundant or absent. For this reason, the ${{\rm E}}_{{fCO}2(s)}$ values were further scaled over time by calculating two scaling factors specific to each LME, one representing the seasonal data coverage (using 3-month running means of the relative data coverage across the seasonal cycle) and another representing the relative annual data coverage (using 5-year running means of the relative data coverage across the timeseries).

The seasonal scaling factor (${{\rm{\varepsilon }}}_{{seas}.}$) was calculated as:

$${{\rm{\varepsilon }}}_{seas.}=\frac{({\sum }_{my=m{y}_{ref}-1}^{m{y}_{ref}+1}{n}_{obs(my)}/{n}_{tot(my)})/MY}{{n}_{obs(my)}/{n}_{tot(my)}}$$

where *my* is the numbered month of the year (1–12), *my*_*ref*_ is the reference month of the year for each time step (1–12), *n*_*obs(my)*_ is the number of grid cells with observations in the corresponding month of the year, *n*_*tot(my)*_ is the total number of available grid cells in the corresponding month of the year, and *MY* is the total number of months considered within the window for each time step. Because January (1) comes after December (12), *my*_*ref*_ − 1 = 12 when *my*_*ref*_ = 1 and *my*_*ref*_ + 1 = 1 when *my*_*ref*_ = 12. The long-term scaling factor (${{\rm{\varepsilon }}}_{{ann}.}$) was calculated as:

$${{\rm{\varepsilon }}}_{ann.}=\frac{({\sum }_{ms=m{s}_{ref}-24}^{m{s}_{ref}+24}{n}_{obs(ms)}/{n}_{tot(ms)})/MS}{{n}_{obs(ms)}/{n}_{tot(ms)}}$$

where *ms* is the numbered month in the full series (1–228), *ms*_*ref*_ is the reference month in the series for each time step (1–228), *n*_*obs(ms)*_ is the number of grid cells with observations in the corresponding month of the series, *n*_*tot(ms)*_ is the total number of available grid cells in the corresponding month, and *MS* is the total number of months considered within the window for each time step. Fewer months were considered within each window near the beginning and end of the time series. Finally, the estimated uncertainty of *f*CO_2(RFR-LME)_ scaled spatially and temporally (i.e., seasonally and annually) was calculated as:

$${{\rm E}}_{{fCO}2(s,t)}={{\rm E}}_{{fCO}2(s)}\times {{\rm{\varepsilon }}}_{{seas}.}\times {{\rm{\varepsilon }}}_{{ann}.}.$$

The window sizes of the scalers were selected to balance data coverage in each time window with realistic periods of time over which observational data may exhibit serial correlations.

Uncertainties in ESPER-estimated *A*_T_ and nutrients were provided by the ESPER algorithms, which estimate uncertainty using a polynomial fit to salinity and depth. The ESPER algorithms are less skillful in the surface ocean where we use them than they are globally across all depths, and the uncertainty estimates are correspondingly greater at shallow depths.

The uncertainty estimates were propagated along with standard estimated total uncertainties in carbonate system constants (see Table 1 in Orr *et al*.^64^) to calculate uncertainty in mapped OA indicators. Gaussian uncertainty propagation was employed, using CO2SYSv3 for MATLAB^56^, which is based on uncertainty propagation code introduced in CO2SYSv2 by Orr *et al*.^64^.

### Validation and evaluation

The skill of the RFR-LME maps was evaluated through comparisons with co-located OA indicators independently calculated from the ship-based GLODAPv2.2022 and CODAP-NA measurements described above. OA indicators were computed at *in situ* temperature from the *A*_T_ and *C*_T_ observations using CO2SYSv3 for MATLAB^56^ and the same equilibrium constants as before. Although the GLODAPv2.2022 and CODAP-NA databases also include pH_T_ and *p*CO_2_ measurements, they are not as widespread as *A*_T_ and *C*_T_ measurements, so we chose to calculate all indicators from *A*_T_ and *C*_T_ for evaluation. Each observation was then co-located with the corresponding RFR-LME grid cell and compared.

In addition, RFR-LME maps were compared to global mapped data products of sea surface *p*CO_2_ obtained from SeaFlux (v2021.04)^47^. Long-term averages of *p*CO_2_ from RFR-LME maps and SeaFlux maps were computed across the overlapping time periods of both products (i.e., 1998–2019). Mapped differences between RFR-LME and each SeaFlux ensemble member, as well as an average across the ensemble, were computed and compared.

Finally, observations of *p*CO_2_ at fixed buoy locations were compared to *p*CO_2_ from RFR-LME data products at grid cells corresponding to those moored buoy observations. For this exercise, special-case RFR-LME maps were created by training RFRs on gridded *f*CO_2(SOCAT)_ data with buoy observations excluded, then using those algorithms to construct the maps. Comparing *p*CO_2_ mapped from datasets both with and without the underlying buoy observations allowed for evaluation of the influence that those seasonally resolved observations have on the fidelity of the *p*CO_2_ reconstruction. *p*CO_2_ values extracted from the mapped SeaFlux datasets were also included in this comparison, allowing for separate evaluation of how the LME-scale, 0.25° × 0.25° monthly reconstructions compare to global 1° × 1° monthly reconstructions.

## Data Records

RFR-LME maps can be accessed through the NOAA National Centers for Environmental Information (NCEI) via the Ocean Carbon and Acidification Data System (OCADS; 10.25921/h8vw-e872)^35^. The dataset is available in NetCDF format on 0.25° × 0.25° spatial grids at monthly timesteps. Each mapped OA indicator and its uncertainty is provided via a separate NetCDF file, along with a reference grid that indicates to which LME each spatial grid cell belongs. Additionally, regional timeseries for CO_2_ partial pressure, calcium carbonate saturation state, and pH are displayed at the NOAA Marine Ecosystem Status website (https://ecowatch.noaa.gov). Average values, trends, seasonal amplitudes, and uncertainty estimates of ocean acidification indicators from RFR-LMEs vary considerably among the regions (Tables 3–6).

**Table 3:** Long-term mean values for OA indicators in each LME.

LME	*p*CO_2_ μatm	*A*_T_ μM	*C*_T_ μM	pH_T_	[H^+^]_T_ nM	RF	Ω_ar_	Ω_ca_	[CO_3_^2−^]_T_ μM
**CCS**	368.4	2197.1	1993.6	8.065	8.63	11.8	2.23	3.50	144.7
**GA**	363.2	2171.6	2010.9	8.065	8.68	13.4	1.78	2.82	116.1
**AI**	395.5	2213.9	2069.8	8.035	9.27	14.2	1.62	2.57	106.2
**EBS**	382.3	2204.6	2060.1	8.049	9.04	14.3	1.62	2.57	106.4
**BS**	301.5	2135.5	2013.6	8.124	7.54	15.3	1.39	2.23	91.1
**NBCS**	331.3	2153.5	2020.2	8.096	8.14	14.8	1.51	2.41	98.8
**NE**	366.2	2273.1	2029.1	8.075	8.46	11.1	2.68	4.14	172.5
**SE**	375.7	2374.6	2039.2	8.072	8.49	9.2	3.69	5.59	234.6
**GM**	381.7	2371.8	2040.9	8.070	8.54	9.3	3.71	5.62	233.4
**CS**	377.1	2320.2	1976.8	8.062	8.68	8.9	3.85	5.79	239.9
**PI**	375.6	2299.6	1970.5	8.063	8.67	9.1	3.68	5.55	229.9
**All**	374.1	2269.9	1999.8	8.063	8.69	10.7	3.01	4.59	190.3

Long-term means are calculated as averages over the monthly timeseries (1998–2022) of area-weighted average indicator values.

**Table 4:** Long-term trends and uncertainties for OA indicators in each LME.

LME	*p*CO_2_	*A*_T_	*C*_T_	pH_T_	[H^+^]_T_	RF	Ω_ar_	Ω_ca_	[CO_3_^2−^]_T_
	μatm/yr	μM/yr	μM/yr	10^−3^/yr	nM·10^−2^/yr	10^−2^/yr	10^−3^/yr	10^−3^/yr	μM·10^−1^/yr
**CCS**	1.8 ± 0.2	0.0 ± 0.5	0.6 ± 0.3	−1.8 ± 0.1	3.5 ± 0.3	1.6 ± 0.5	−4.7 ± 1.6	−7.6 ± 2.4	−3.2 ± 1.0
**GA**	1.1 ± 0.1	−0.4 ± 0.3	−0.1 ± 0.5	−1.2 ± 0.1	2.3 ± 0.3	1.0 ± 1.4	−2.3 ± 3.1	−3.8 ± 4.5	−1.6 ± 1.8
**AI**	0.9 ± 0.1	−0.3 ± 0.2	−0.1 ± 0.1	−0.9 ± 0.1	1.9 ± 0.3	0.4 ± 0.3	−1.1 ± 0.6	−1.8 ± 0.9	−0.8 ± 0.4
**EBS**	0.9 ± 0.1	−0.3 ± 0.1	−0.2 ± 0.1	−0.8 ± 0.1	1.8 ± 0.3	0.0 ± 0.3	0.3 ± 0.8	0.3 ± 1.2	0.1 ± 0.5
**BS**	1.1 ± 0.1	0.6 ± 0.4	0.9 ± 0.3	−1.4 ± 0.2	2.4 ± 0.3	1.7 ± 0.5	−2.2 ± 1.1	−3.7 ± 1.7	−1.5 ± 0.7
**NBCS**	0.3 ± 0.1	0.8 ± 0.4	0.5 ± 0.4	−0.2 ± 0.2	0.5 ± 0.3	−0.9 ± 0.4	2.6 ± 1.0	3.9 ± 1.5	1.7 ± 0.6
**NE**	1.3 ± 0.1	1.0 ± 0.5	0.8 ± 0.3	−1.2 ± 0.1	2.3 ± 0.2	−0.3 ± 0.5	2.3 ± 3.2	2.7 ± 4.7	1.3 ± 2.2
**SE**	1.8 ± 0.2	0.3 ± 0.1	1.0 ± 0.1	−1.7 ± 0.2	3.4 ± 0.4	1.1 ± 0.1	−6.5 ± 0.7	−10.5 ± 1.0	−4.4 ± 0.4
**GM**	1.4 ± 1.2	0.1 ± 0.1	0.6 ± 0.2	−1.3 ± 1.1	2.7 ± 2.2	0.8 ± 0.2	−5.0 ± 1.3	−8.0 ± 1.9	−3.3 ± 0.9
**CS**	1.2 ± 0.1	0.7 ± 1.9	1.0 ± 1.4	−1.1 ± 0.1	2.2 ± 0.2	0.5 ± 0.1	−2.9 ± 3.3	−4.9 ± 4.6	−1.9 ± 2.2
**PI**	1.6 ± 0.1	−0.3 ± 0.8	0.4 ± 0.8	−1.6 ± 0.1	3.1 ± 0.2	1.0 ± 0.1	−7.3 ± 1.1	−11.5 ± 1.6	−5.0 ± 0.8
**All**	1.4 ± 0.4	−0.2 ± 0.5	0.4 ± 0.6	−1.4 ± 0.4	2.7 ± 0.9	0.8 ± 0.9	−0.5 ± 0.3	−0.8 ± 0.5	−3.2 ± 1.9

Trends and trend uncertainties are determined by fitting a linear least-squares model with an intercept, trend, and annual and semi-annual harmonics to monthly area-weighted average indicator values. Area-weighted averages are calculated using a consistent fraction of ice-free cells for each month in each region, even though in reality some years have less ice coverage than others. Uncertainties are calculated by scaling the standard error on the trend by the effective degrees of freedom, determined from the decorrelation timescale of residual values.

**Table 5:** Seasonal amplitudes for OA indicators in each LME.

LME	*p*CO_2_ μatm	*A*_T_ μM	*C*_T_ μM	pH_T_	[H^+^]_T_ nM	RF	Ω_ar_	Ω_ca_	[CO_3_^2−^]_T_ μM
**CCS**	12.3	11.0	35.1	0.011	0.21	1.0	0.33	0.49	19.5
**GA**	95.3	24.8	109.8	0.107	2.12	3.6	0.85	1.32	54.2
**AI**	77.9	14.2	83.2	0.083	1.76	3.1	0.66	1.02	42.3
**EBS**	137.2	42.5	131.5	0.147	2.96	4.0	0.84	1.32	54.1
**BS**	56.0	205.8	206.6	0.070	1.19	1.4	0.23	0.37	15.0
**NBCS**	110.8	87.3	174.1	0.142	2.49	4.3	0.84	1.33	54.2
**NE**	56.5	36.1	103.5	0.055	1.06	2.4	0.95	1.36	54.0
**SE**	59.9	15.8	43.9	0.065	1.26	0.6	0.45	0.57	21.1
**GM**	49.6	2.5	45.9	0.051	1.01	0.8	0.72	0.96	34.7
**CS**	32.8	68.1	49.1	0.039	0.77	0.1	0.16	0.25	13.2
**PI**	23.8	1.7	11.3	0.026	0.50	0.2	0.16	0.20	7.2
**All**	12.3	20.1	44.2	0.014	0.27	1.0	0.29	0.42	16.2

Seasonal amplitudes are calculated from the annual sine and cosine component amplitudes of a linear least-squares model with an intercept, trend, and annual and semi-annual harmonics fit to area-weighted average indicator values.

**Table 6:** Average uncertainties for OA indicators in each LME.

LME	*p*CO_2_ μatm	*A*_T_ μM	*C*_T_ μM	pH_T_	[H^+^]_T_ nM	RF	Ω_ar_	Ω_ca_	[CO_3_^2−^]_T_ μM
**CCS**	20.5	15.6	18.4	0.022	0.47	0.40	0.17	0.26	7.9
**GA**	24.4	17.8	19.9	0.026	0.56	0.54	0.14	0.23	7.3
**AI**	20.6	13.6	15.4	0.021	0.48	0.49	0.12	0.19	5.7
**EBS**	18.1	16.2	17.3	0.020	0.43	0.50	0.12	0.19	5.7
**BS**	58.9	33.1	45.9	0.070	1.36	1.43	0.27	0.43	16.7
**NBCS**	106.2	27.8	56.8	0.097	2.38	2.15	0.39	0.63	25.0
**NE**	23.4	14.5	19.2	0.025	0.51	0.42	0.20	0.31	9.3
**SE**	10.8	8.3	12.3	0.013	0.26	0.16	0.22	0.33	8.0
**GM**	24.1	12.4	20.4	0.024	0.48	0.29	0.27	0.42	12.3
**CS**	3.4	11.5	11.6	0.008	0.16	0.11	0.21	0.31	6.5
**PI**	2.8	12.5	12.1	0.008	0.15	0.11	0.20	0.30	6.3
**All**	11.6	13.8	15.2	0.015	0.31	0.27	0.18	0.28	7.1

Uncertainties are determined by filtering and scaling *f*CO_2_ error estimates, pairing those with *A*_T_ uncertainties from ESPER estimates, and propagating those through CO_2_ system calculations.

**Fig. 4:**
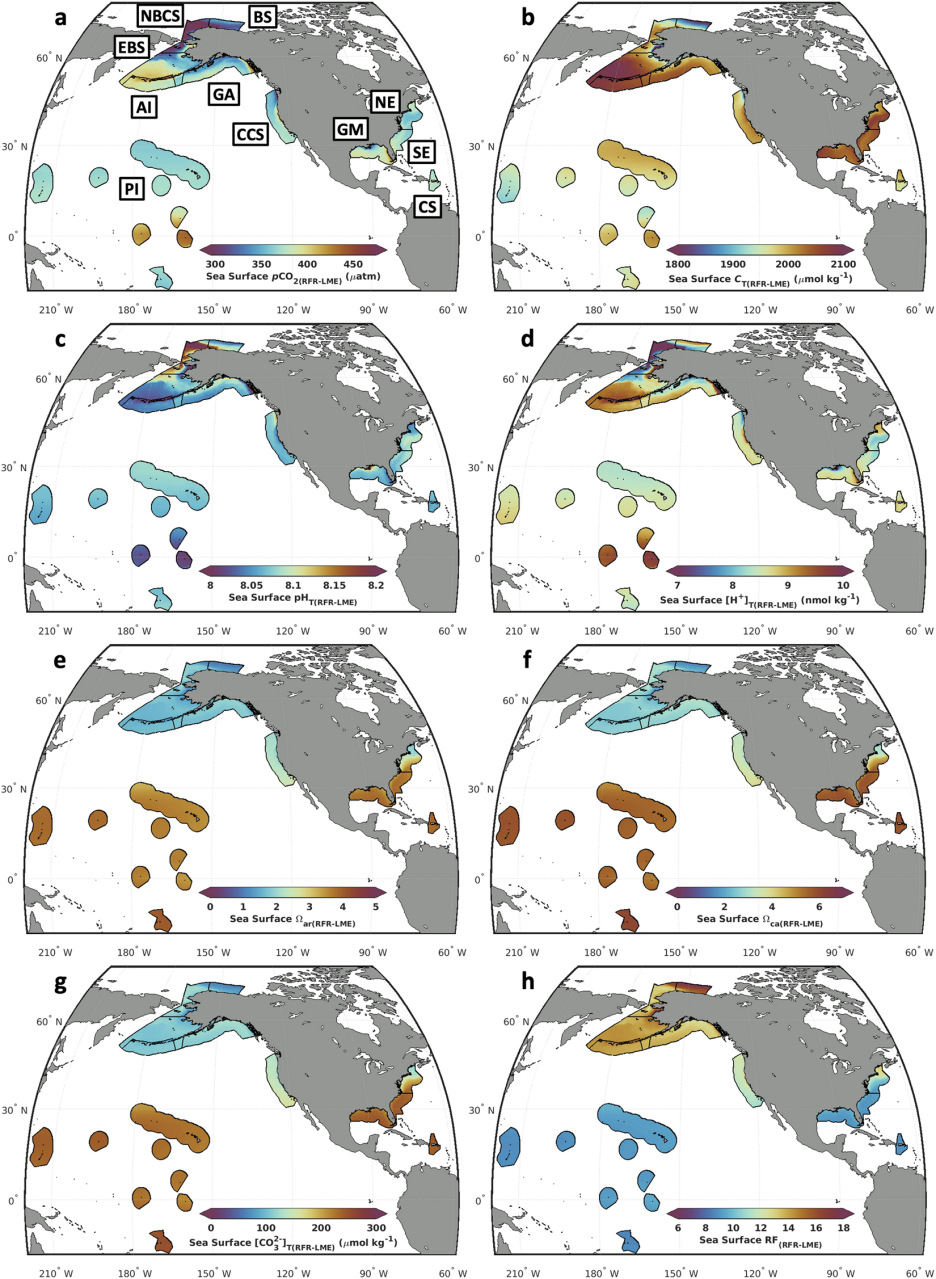
Long-term means of RFR-LME mapped OA indicators. Mapped averages of (**a**) *p*CO_2(RFR-LME)_, (**b**) *C*_T(RFR-LME)_, (**c**) pH_T(RFR-LME)_, (**d**) [H^+^]_T(RFR-LME)_, (**e**) Ω_ar(RFR-LME)_, (**f**) Ω_ca(RFR-LME)_, (**g**) [CO_3_^2−^]_T(RFR-LME)_, and (**h**) RF_(RFR-LME)_ over the timeseries (1998–2022) within each LME.

Long-term means (Table 3; Fig. 4) allow for the description of LME-scale patterns in surface ocean carbonate chemistry. Tropical LMEs (PI and CS) are characterized most notably by high carbonate ion parameters ([CO_3_^2−^]_T_, Ω_ar_, and Ω_ca_) and low RF values. Within this pair, the CS can be described as more acidified (higher *p*CO_2_ and lower pH_T_) but better buffered (lower RF and higher *A*_T_/*C*_T_ ratio). Subtropical Atlantic LMEs (GM and SE) also have high carbonate ion parameters ([CO_3_^2−^]_T_, Ω_ar_, and Ω_ca_) and low RF values. Compared to the Tropical LMEs however, Subtropical Atlantic LMEs have higher *C*_T_ and *A*_T_ values, although *A*_T_/*C*_T_ ratios and therefore RFs are similar between the two groups. Temperate and subarctic coastal LMEs (CCS, GA, and NE) can generally be considered intermediate in all parameters: *p*CO_2_, pH, carbonate ion parameters, *C*_T_, *A*_T_, and RF. Within the group, the GA has the highest RF and lowest carbonate ion parameters, the NE has the lowest RF and highest carbonate ion parameters, and the CCS is between the two. Subarctic North Pacific LMEs (AI and EBS) are characterized by high *C*_T_, *p*CO_2_, and RF; and low pH_T_ and carbonate ion parameters. Arctic LMEs (NBCS and BS) are characterized by high pH_T_ and RF; and low *A*_T_, *p*CO_2_, and carbonate ion parameters.

**Fig. 5:**
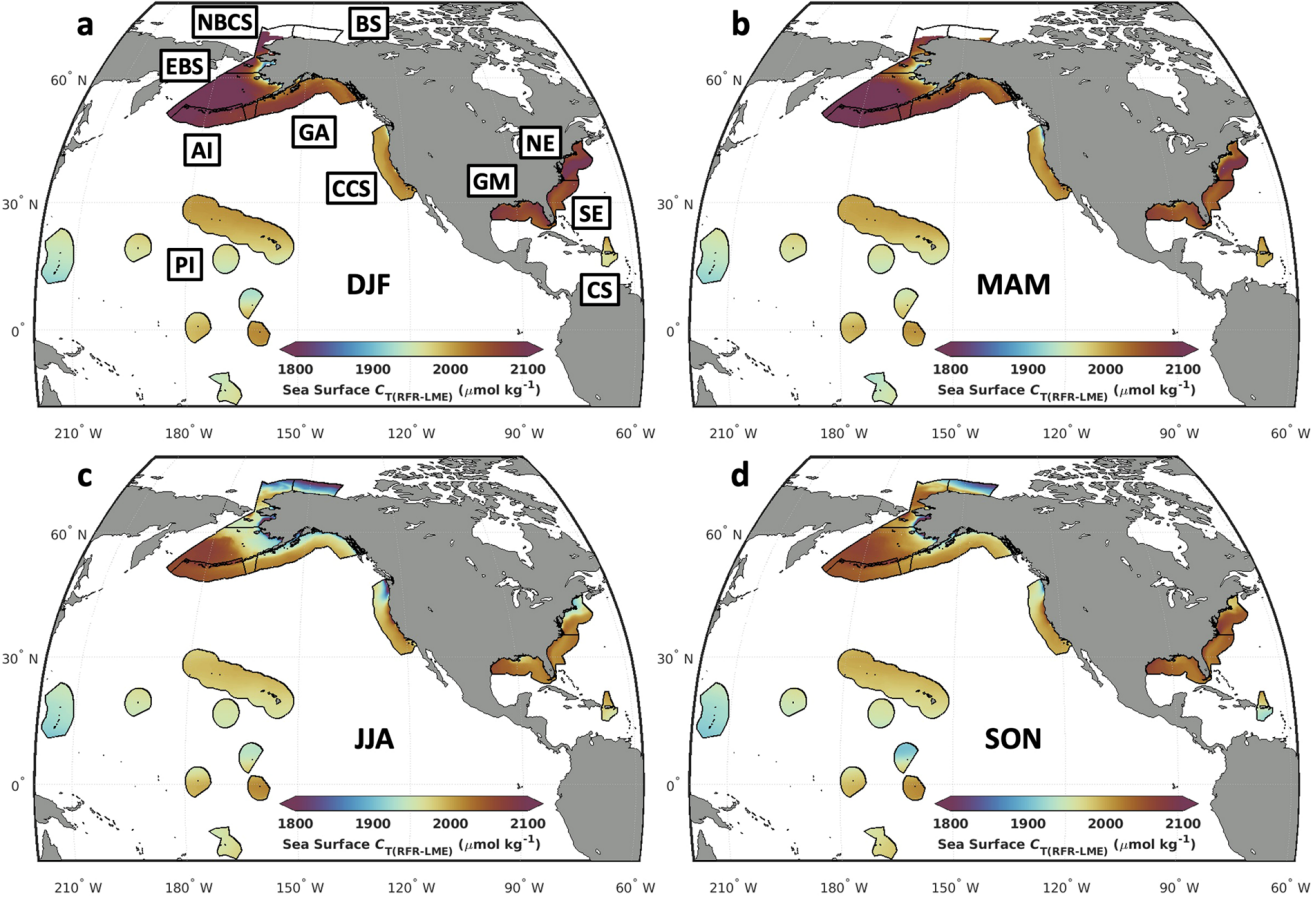
Seasonal means of *C*_T(RFR-LME)_. Mapped averages of *C*_T(RFR-LME)_, in the northern hemisphere (**a**) winter (DJF), (**b**) spring (MAM), (**c**) summer (JJA), and (**d**) fall (SON) over the timeseries (1998–2022) within each LME.

Spatial variability in OA indicators is evident within each LME and throughout the seasonal cycle (Fig. 5). For example, the CCS develops a strong dipole in the summer (June/July/August; Fig. 5c), with low *C*_T_ off the coast in the northern *C*_T_ and high *C*_T_ off the coast in the central CCS. This dipole becomes much weaker in the winter (December/January/February; Fig. 5a). Similarly, relatively low *C*_T_ occurs off the coast in the northern NE region in the summer but disappears in the winter (Fig. 5c). The southern continental Alaskan coastline exhibits low *C*_T_, especially nearshore in the summer, whereas the northern Alaskan coastline is relatively higher in *C*_T_ than nearby offshore waters in the Arctic Ocean. A band of relatively low *C*_T_ is evident from about 10° to 20° N in the PI region, between higher *C*_T_ in the equatorial Pacific and North Pacific subtropical gyre, a feature that has appeared in other sea surface *C*_T_ data products^6^.

**Fig. 6:**
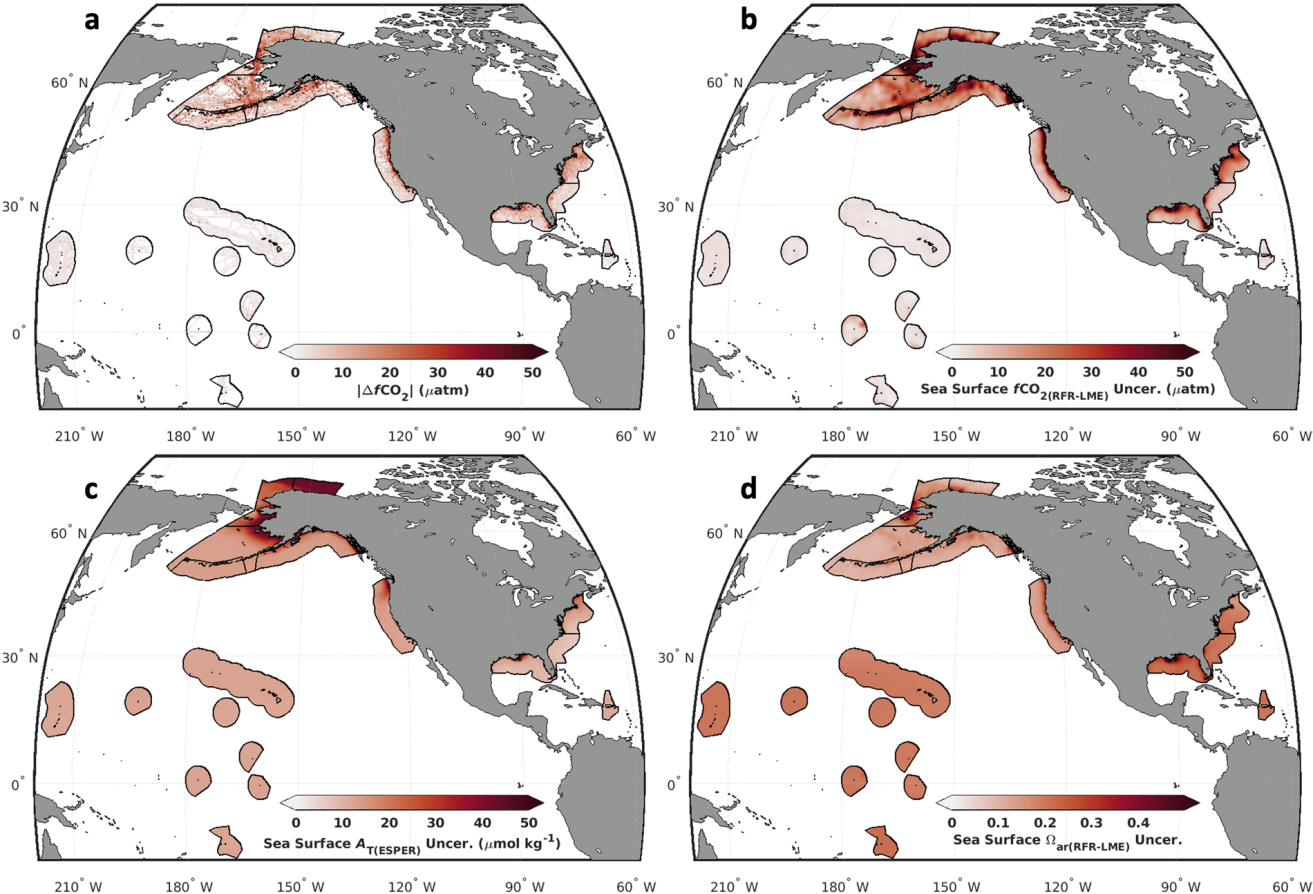
*k*-fold cross-validated differences between estimated and measured *f*CO_2_ and scaled uncertainty in *f*CO_2(RFR-LME)_, *A*_T(ESPER)_, and Ω_ar(RFR-LME)_. (**a**) Absolute differences (|Δ*f*CO_2_|) between *f*CO_2(RFR-LME-kFold)_ and *f*CO_2(SOCAT)_ calculated via a *k*-fold cross-validation approach compared to (**b**) long-term average uncertainty in *f*CO_2(RFR-LME-kFold)_, calculated and scaled according to the procedure in the *Uncertainty estimation* section. Long-term average uncertainty in (**c**) *A*_T(ESPER)_ and (**d**) Ω_ar(RFR-LME)_ are also shown.

Mapped indicator uncertainties (see Fig. 6) are served alongside RFR-LME maps^35^, providing a resource for evaluating uncertainty in OA indicator values at a given location. Area-weighted mean *u*[*p*CO_2(RFR-LME)_] was 12.0 μatm across the entire domain, *u*[pH_T(RFR-LME)_] was 0.015, and *u*[Ω_ar(RFR-LME)_] was 0.18. These domain-wide means are influenced by the large area and low uncertainties in the Pacific Islands region; individual LME uncertainties, particularly in Artic and Subarctic LMEs, may be considerably larger. Spatial patterns of uncertainties also differ for different OA indicators. For example, *u*[Ω_ar(RFR-LME)_] tends to be relatively high in the tropical LMEs (Fig. 6d), where Ω_ar(RFR-LME)_ is also high (Fig. 4e); on the other hand, *u*[*p*CO_2(RFR-LME)_] is extremely low in tropical the LMEs (Fig. 6b).

Uncertainty values reflect not only uncertainty in the RFR predictions, but also uncertainty introduced by interpolating over spatial and temporal gaps in observational coverage. Average uncertainty values for each LME are presented alongside OA indicator timeseries on the NOAA NaMES website. Importantly, the uncertainty values provided in Table 6 and on the NaMES website represent weighted means of grid-cell-level uncertainties rather than uncertainties corresponding to region-wide averages, which may or may not be smaller due to cancelling errors that are removed by areal averaging or larger due to inadequacies of our spatiotemporal scaling approach for representing uncertainties in under-sampled times and locations.

## Technical Validation

### Data-based validation

A *k*-fold cross-validation approach was used to assess the skill of the *f*CO_2_ estimates and subsequent OA indicator calculations. Region-wide error statistics for each of the eleven LMEs (before the spatial and temporal scaling) indicate that *f*CO_2(RFR-LME-kFold)_ values are centered around (mean and median errors all close to zero) and tend to correlate closely with (nine of the eleven *R*^2^ values are 0.8 or greater) the measured values of *f*CO_2(SOCAT)_ (Table 7). Root mean square errors (RMSEs) are generally about three times larger than median absolute errors, indicating error populations with long tails of a few particularly large errors. When viewed spatially (Fig. 6a), absolute differences (|Δ*f*CO_2_|) between *f*CO_2(RFR-LME-kFold)_ and *f*CO_2(SOCAT)_ are greatest near the coast and in the North Pacific and Arctic, and smallest in the open ocean and in the tropics and subtropics. High |Δ*f*CO_2_| values tend to correlate with areas of high background variability in *f*CO_2(SOCAT)_ (Fig. 2b), emphasizing that the RFR algorithms may struggle to capture extreme values, which is consistent with the aforementioned long-tailed error populations.

**Table 7:** Error statistics of *f*CO_2_ predicted by *k*-fold cross-validation algorithms (*f*CO_2(RFR-LME-kFold)_) compared to *f*CO_2_ from SOCAT observations (*f*CO_2(SOCAT)_).

LME	Mean Δ*f*CO_2_	Mean |Δ*f*CO_2_|	Median Δ*f*CO_2_	Median |Δ*f*CO_2_|	IQR	RMSE	*R*^2^
**CCS**	−0.51	21.48	0.61	8.47	16.85	44.10	0.67
**GA**	0.11	14.03	0.16	7.66	15.31	23.95	0.89
**AI**	0.02	13.44	0.64	6.52	13.10	26.11	0.76
**EBS**	0.79	14.81	0.67	9.33	18.53	22.40	0.92
**BS**	−0.35	8.68	−0.57	4.64	9.24	14.40	0.91
**NBCS**	−1.04	19.14	0.05	11.93	23.85	29.95	0.88
**NE**	−0.11	15.58	0.39	10.86	21.70	22.89	0.81
**SE**	−0.08	8.01	0.47	4.14	8.30	16.82	0.82
**GM**	−0.07	13.82	−0.01	6.00	12.03	29.08	0.75
**CS**	−0.05	3.68	0.24	2.46	4.89	5.41	0.93
**PI**	−0.02	2.37	0.09	1.62	3.24	3.56	0.98
**Average**	−0.10	12.24	0.25	6.68	13.34	21.53	0.85

The *k*-fold cross-validation procedure performed within each LME is described in the *Machine learning regressions* section. Statistics shown include the mean and absolute mean Δ*f*CO_2_ (*f*CO_2(RFR-LME-kFold)_ − *f*CO_2(SOCAT)_), median and absolute median Δ*f*CO_2_, interquartile range (IQR) of Δ*f*CO_2_, root mean square error (RMSE) of Δ*f*CO_2_, and Pearson’s correlation coefficient (*R*^2^) between and *f*CO_2(RFR-LME-kFold)_ and *f*CO_2(SOCAT)_.

### Comparison to global trends

**Fig. 7:**
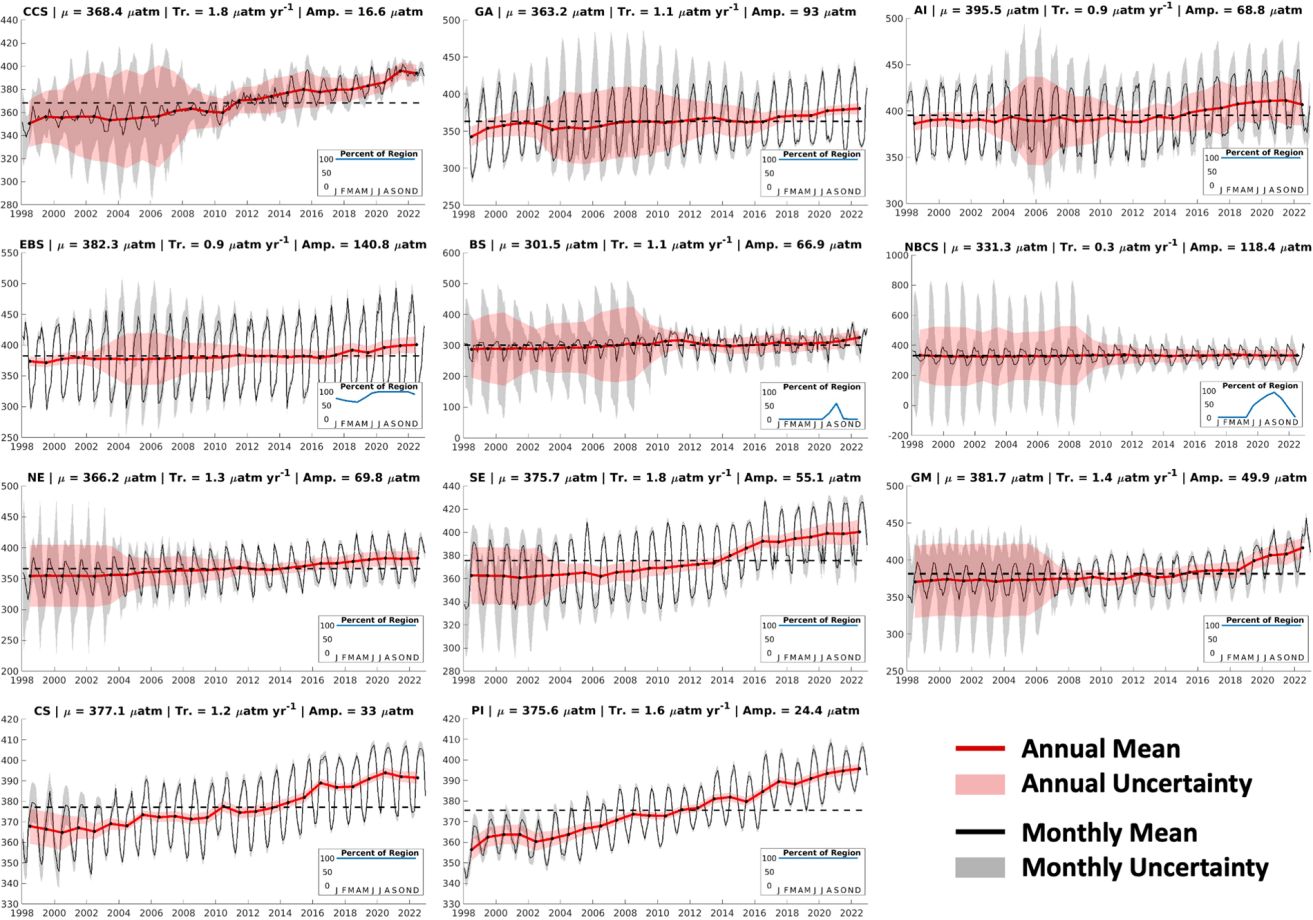
*p*CO_2_ timeseries for the eleven U.S. LMEs considered in this study. Area-weighted annual (red) and monthly (black) means are presented along with envelopes of uncertainty. Uncertainties are calculated by scaling *k*-fold cross-validated uncertainties spatially with a two-dimensional low-pass filter, then temporally according to long-term data coverage (5-year running windows) and seasonal data coverage (3-month running windows). Average values across the timeseries are indicated by dotted lines. Note the different *y*-axis for each LME. For many LMEs, uncertainties are larger near the beginning of the timeseries, when SOCAT observations are less dense. Inset within each timeseries plot is a figure showing the percent of the LME represented across the seasonal cycle by grid cells that remain ice-free across the entire timeseries; these are the grid cells used to compute monthly and annual means.

**Fig. 8:**
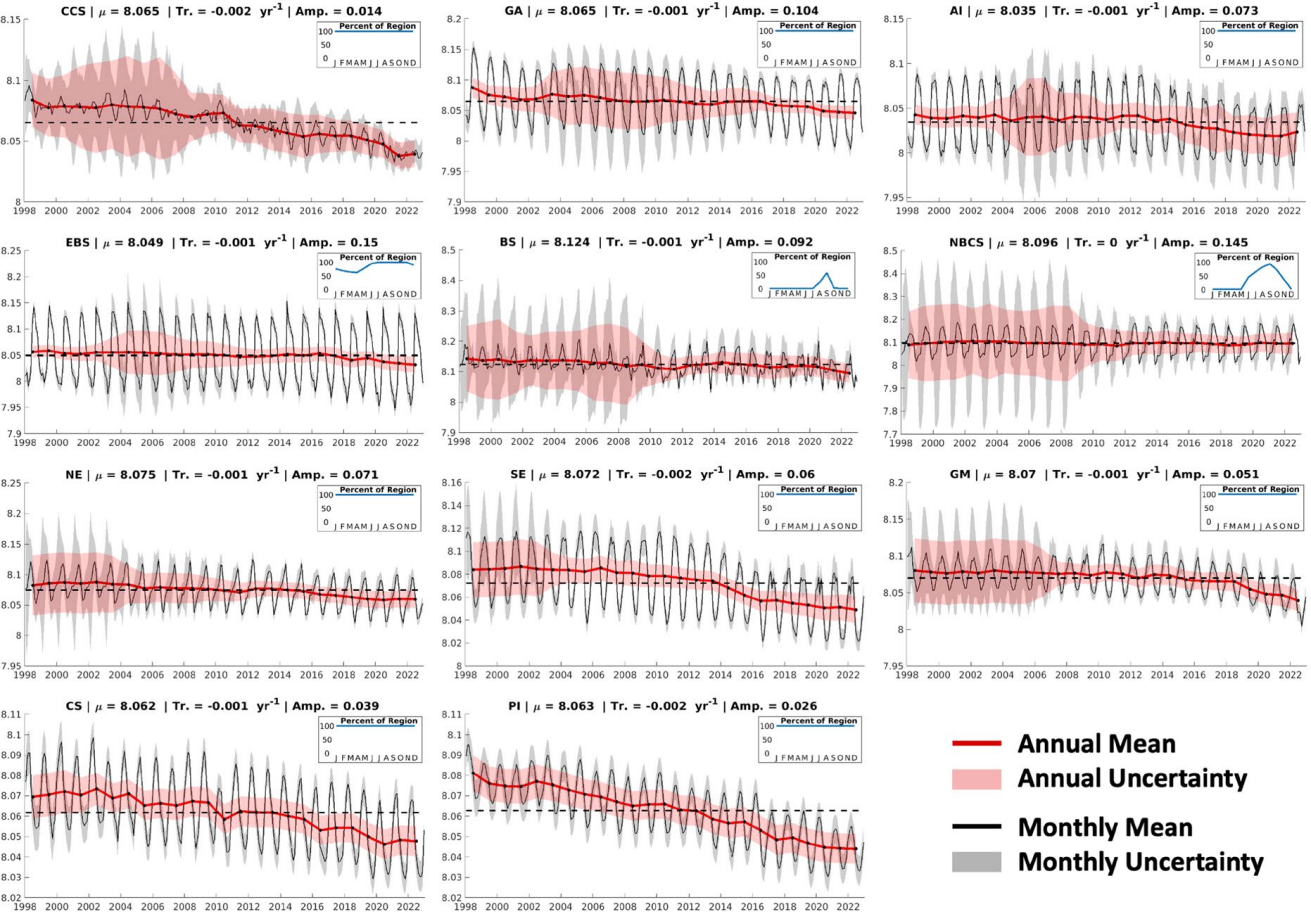
pH_T_ timeseries for the eleven U.S. LMEs considered in this study. Same as Fig. 7, except for pH_T_.

**Fig. 9:**
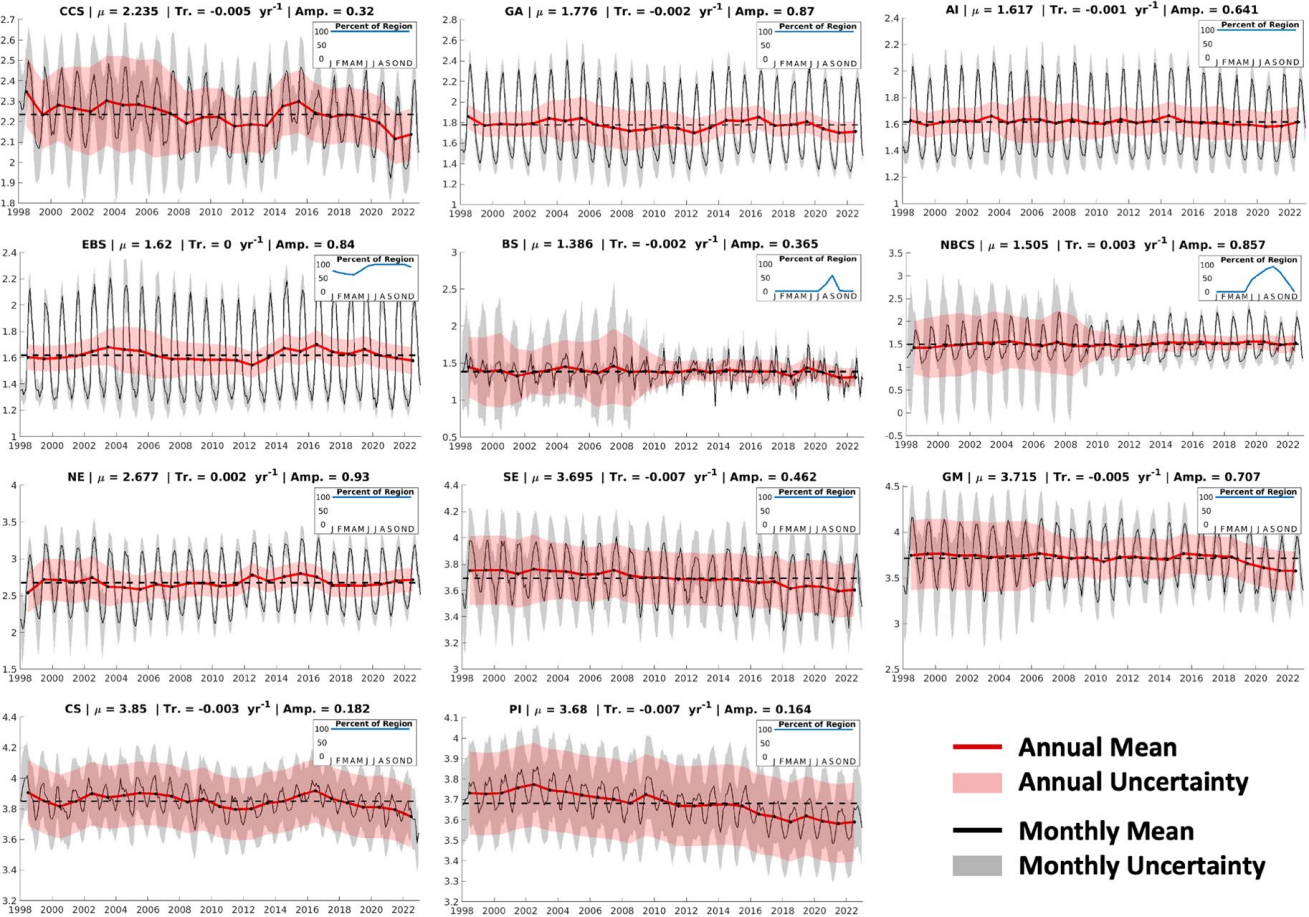
Ω_ar_ timeseries for the eleven U.S. LMEs considered in this study. Same as Fig. 7, except for Ω_ar_.

RFR-LME indicator timeseries (1998–2022) represent spatially weighted annual averages of OA indicators computed from RFR-LME maps. Increasing *p*CO_2(RFR-LME)_ and decreasing pH_T(RFR-LME)_ are observed in each LME (Figs. 7, 8) — trends that are strongly influenced by anthropogenic CO_2_ uptake and amplified by ocean warming (Table 8). Ω_ar(RFR-LME)_ decreases in many (but not all) LMEs over 1998–2022 (Fig. 9), as Ω_ar_ decline is driven by anthropogenic CO_2_ uptake as well, but moderated by ocean warming and also influenced by changes in SSS (Table 8). Trends in OA indicators across U.S. LMEs (Table 4) can be compared with global trends of about + 1.5 μatm yr^–1^ for *p*CO_2_ (+0.3 to +1.8 μatm yr^–1^ for RFR-LMEs), +0.9 μmol kg^–1^ yr^–1^ for *C*_T_ (–0.2 to +1.0 μmol kg^–1^ yr^–1^ for RFR-LMEs), –1.7·10^–3^ units yr^–1^ for pH_T_ (–1.8·10^–3^ to –0.2·10^–3^ yr^–1^ for RFR-LMEs), and –7.0·10^–3^ yr^–1^ for Ω_ar_^1,4,6,10^ (–7.3·10^–3^ to +2.6·10^–3^ yr^–1^ for RFR-LMEs).

**Table 8:** Long-term mean values, trends, and seasonal amplitudes for temperature and salinity in each LME.

LME	Means		Trends		Amplitudes	
	Temp. °C	Sal	Temp. °C/yr	Sal yr^−1^	Temp.	Sal
CCS	14.0	32.6	0.04 ± 0.03	0.00 ± 0.01	4.9	0.3
GA	7.9	32.0	0.04 ± 0.04	−0.01 ± 0.01	7.9	0.6
AI	6.2	32.7	0.04 ± 0.01	−0.01 ± 0.00	6.7	0.2
EBS	5.5	32.5	0.05 ± 0.06	0.00 ± 0.00	6.8	0.9
BS	−0.4	30.2	0.01 ± 0.01	0.01 ± 0.01	3.8	4.3
NBCS	2.1	30.9	0.04 ± 0.01	0.02 ± 0.01	9.2	1.7
NE	16.3	34.0	0.07 ± 0.01	0.02 ± 0.01	13.5	0.8
SE	24.8	36.2	0.03 ± 0.01	0.01 ± 0.00	7.5	0.3
GM	25.5	35.0	0.02 ± 0.01	0.01 ± 0.00	8.9	0.9
CS	27.8	35.3	0.02 ± 0.01	0.01 ± 0.03	2.7	1.2
PI	26.7	35.0	0.03 ± 0.01	−0.01 ± 0.01	2.8	0.0

Long-term means are calculated as averages over the monthly timeseries (1998–2022) of area-weighted average values; trends and trend uncertainties are determined by fitting a linear least-squares model with an intercept, trend, and annual and semi-annual harmonics to monthly area-weighted average values; and seasonal amplitudes are calculated from the annual sine and cosine component amplitudes of the linear least-squares model.

It is important to note that, for some of the Arctic and subarctic LMEs that are characterized by high seasonal ice coverage, these trends are driven by primarily summertime OA indicator values (see inset plots in Figs. 7–9). This limitation, along with the fact that these timeseries are relatively short (25 years) and regionally limited, can explain divergence in some specific cases from the global trends.

### Comparison to discrete shipboard data

The RFR-LME fields presented in this work are constructed using surface CO_2_ measurements from shipboard flow-through analyzers. This automated observational approach allows for the collection of high spatial and temporal resolution observations of surface ocean carbonate chemistry. Discrete bottle measurements of carbonate chemistry parameters represent another approach for monitoring ocean acidification. The discrete approach allows for high-quality observations throughout the water column. Here we take near-surface discrete bottle measurements of *A*_T_ and *C*_T_ from GLODAPv2.2022^51^ and CODAP-NA^52^, use those measurements to calculate OA indicators, and compare those calculated values with mapped surface OA indicators from RFR-LMEs.

**Fig. 10:**
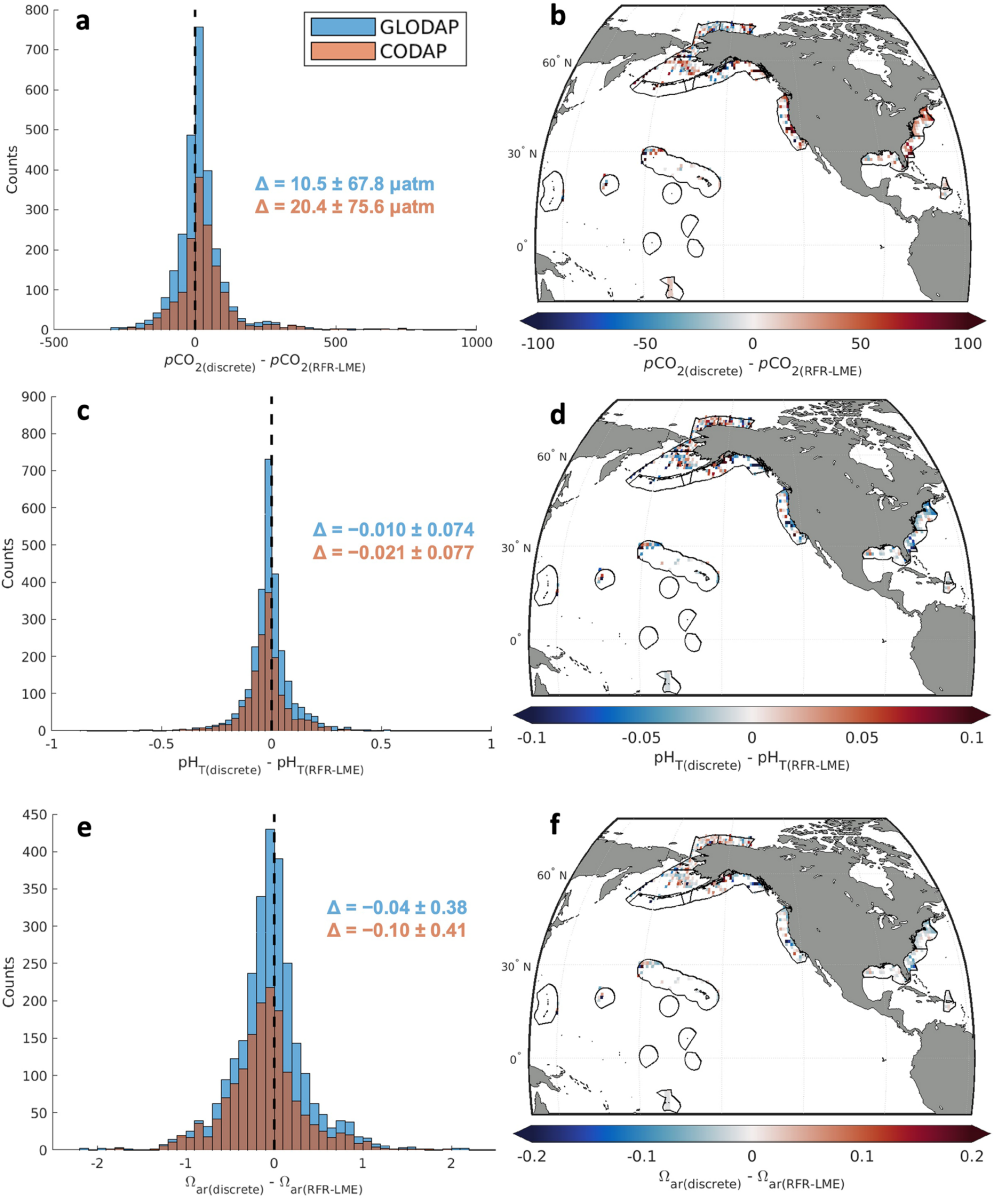
Comparisons between OA indicators retrieved from RFR-LME maps and those calculated from discrete observations. (**a,c,e**) Histograms showing differences between calculations of (**a**) *p*CO_2_, (**c**) pH_T_, and (**e**) Ω_ar_ from discrete surface (depth ≤ 10 m) observations of *A*_T_ and *C*_T_ and values of the same OA indicators from RFR-LME maps. Discrete observations that fall within the boundaries of LMEs were obtained from the GLODAPv2.2022^51^ and CODAP-NA^52^ data products. Error statistics shown represent the median errors and the interquartile ranges of errors for each comparison. (**b,d,f**) Mapped differences between calculations of (**b**) *p*CO_2_, (**d**) pH_T_, and (**f**) Ω_ar_ from discrete surface (depth ≤ 10 m) observations of *A*_T_ and *C*_T_ and values of the same OA indicators from RFR-LME maps. Discrete differences are binned into 1° × 1° grid cells for this map.

RFR-LME indicator values are generally in good agreement with calculations from discrete bottle measurements (Fig. 10). Compared to the *k*-fold-validation-based uncertainty estimates (Table 6), a greater spread (i.e. larger IQRs) in the differences between GLODAP/CODAP and RFR-LME values is expected in this exercise for two reasons. First, uncertainty stemming from CO_2_ system calculations will contribute to the spread (e.g., Orr *et al*.^64^), since GLODAP/CODAP indicators values are calculated from *A*_T_ and *C*_T_ and RFR-LME indicator values are calculated from *f*CO_2_ and *A*_T_. As an example, average propagated uncertainties for GLODAP/CODAP calculations using standard measurement errors for *A*_T_ and *C*_T_ (2 μmol kg^−1^ for both) and for equilibrium constants^64^ were calculated as 12.0 μatm for *p*CO_2_, 0.014 for pH_T_, and 0.11 for Ω_ar_. In addition, the two datasets are fundamentally different in their spatiotemporal resolution. RFR-LME grid cells represent averages for large swaths of the surface ocean over a monthly timestep, whereas shipboard measurements are appropriate for a distinct point in space at a distinct time. This spatiotemporal mismatch is especially noteworthy in the coastal ocean where diurnal and other sub-monthly modes of variability operate over spatial scales much finer that 0.25 degrees of latitude or longitude. The calculations from bottle measurements also tend to indicate higher *p*CO_2_ and therefore lower pH_T_ and Ω_ar_. These offsets between the two datasets may be partly related to inconsistencies in carbonate chemistry calculations, whereby calculations from *A*_T_ and *C*_T_ at most surface conditions tend to produce lower pH_T_ (and higher *p*CO_2_) values than corresponding measurements of those properties^65,66^.

### Comparison to moored buoy time series data

Timeseries of *p*CO_2_ from fixed grid cells of RFR-LME maps and RFR-LME maps constructed without moored buoy observations (RFR-LME-NM) were compared to *p*CO_2_ observations at fixed buoy locations that were extracted from the SOCATv2023 database and aggregated in monthly bins. This provides a test of the capacity of RFR-LMEs to reproduce monthly variability in validation measurements that were withheld from training, and can be considered an assessment of the RFR-LME skill with monthly variability generally. Mapped global data products of surface ocean carbonate chemistry obtained from SeaFlux^27,47^ were also compared to the moored buoy observations.

**Fig. 11:**
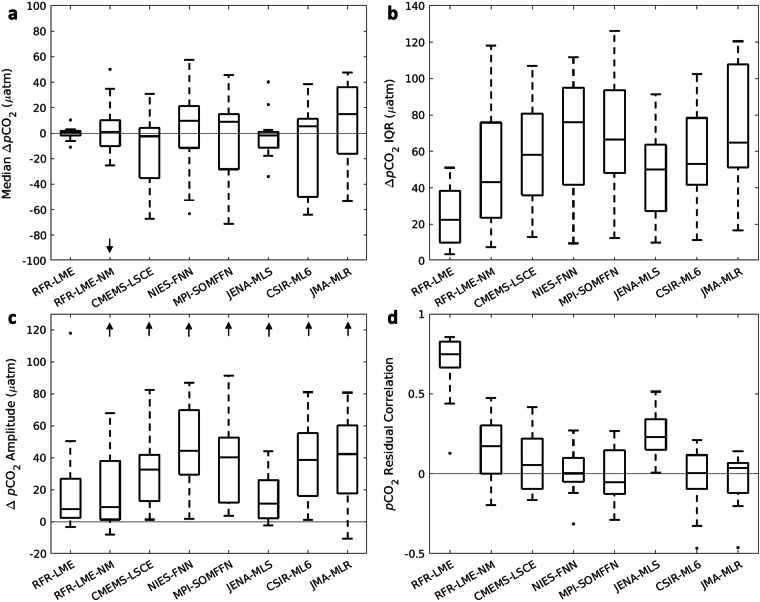
Summarized differences between monthly binned moored buoy *p*CO_2_ observations and mapped *p*CO_2_ data products. (**a**) Medians and (**b**) interquartile ranges of differences, (**c**) differences in seasonal amplitudes, and (**d**) correlations of residual values (after removing the trend and seasonal cycle) between binned moored buoy *p*CO_2_ observations and mapped *p*CO_2_ data products. Each of these statistics are shown as boxplots representative of all 14 mooring sites compared to each mapped product, where the boxes extend from the 25^th^ to 75^th^ percentile, the center line shows the median of the data, the whiskers extend to the most extreme data points not considered outliers, and dots denote outliers (arrows denote where outliers do not appear within the axis limits). RFR-LME is the product described in this work and RFR-LME-NM is the constructed using the same method but without moored buoy observations. References for the SeaFlux mapped products are provided in the *Data sources* section.

Differences between moored buoy observations and mapped products (Table 9; Fig. 11) suggest that timeseries extracted from our regionally focused RFR-LME maps more meaningfully reflect observed *p*CO_2_ than those from mapped global products. Like RFR-LME, most of these alternative products were trained from versions of SOCAT that include the buoy observations. The average median ( ± IQR) Δ*p*CO_2_ (*p*CO_2(moor.)_ – *p*CO_2(grid)_) was 0.1 ± 23.6 μatm for RFR-LME and increased to −13.0 ± 49.2 μatm for the RFR-LME-NM product, which excluded these observations from the training data. These increased error statistics emphasize the value of moored buoy observations for the surface CO_2_ observing system. Still, all but one (JENA-MLS; Δ*p*CO_2_ = −2.2 ± 48.7) of the mapped data products from SeaFlux exhibited more variability in their differences from buoy observations than even the version of RFR-LME constructed without moored buoy observations. JENA-MLS may perform better at representing *p*CO_2_ at these mooring sites because it explicitly models mixed layer fluxes and processes rather than relying on empirical relationships learned from large sets of data.

**Table 9:** Medians and interquartile ranges (μatm) of comparisons between moored buoy observations and corresponding grid cells from mapped monthly sea surface *p*CO_2_ data products.

Buoy	RFR-LME	RFR-LME-NM	CMEMS-LSCE	NIES-FNN	MPI-SOMFFN	JENA-MLS	CSIR-ML6	JMA-MLR
**La Parguera**	1.8 ± 6.1	34.8 ± 23.5	30.7 ± 31.9	21.4 ± 41.6	30.8 ± 32.0	22.4 ± 25.0	27.6 ± 32.7	36.1 ± 34.6
**WHOTS**	3.1 ± 4.6	9.9 ± 7.8	1.8 ± 12.9	10.4 ± 9.5	6.2 ± 12.3	−0.2 ± 9.9	6.0 ± 11.2	6.0 ± 21.5
**Cheeca Rocks**	−11.0 ± 51.0	−25.4 ± 75.8	−7.3 ± 105.4	−11.6 ± 101.9	−7.0 ± 92.1	−2.5 ± 68.6	−8.6 ± 102.4	−12.1 ± 107.7
**Gray’s Reef**	−2.7 ± 23.2	0.8 ± 51.3	12.1 ± 55.5	−4.0 ± 71.0	14.8 ± 66.3	1.0 ± 57.9	4.6 ± 52.4	17.3 ± 60.2
**CCE1**	1.1 ± 3.4	1.1 ± 7.4	0.8 ± 15.6	14.5 ± 25.6	3.9 ± 13.2	−1.5 ± 15.7	4.4 ± 16.9	14.9 ± 16.5
**CCE2**	1.4 ± 14.2	4.6 ± 36.0	2.1 ± 35.9	9.3 ± 41.7	11.4 ± 52.5	2.7 ± 33.3	11.2 ± 41.6	35.6 ± 51.3
**Gulf of Maine**	0.2 ± 17.8	−10.1 ± 32.2	−5.6 ± 43.3	12.8 ± 45.9	12.5 ± 64.8	−2.2 ± 50.1	7.4 ± 53.8	39.7 ± 53.8
**CB-06**	10.3 ± 42.7	50.1 ± 83.5	4.0 ± 89.0	33.2 ± 87.0	45.6 ± 99.8	−14.9 ± 63.6	38.6 ± 99.4	39.3 ± 120.4
**Cape Elizabeth**	1.8 ± 21.8	10.2 ± 50.0	−35.6 ± 77.5	2.5 ± 90.7	−28.3 ± 93.6	−11.4 ± 55.0	−50.0 ± 75.6	−16.0 ± 93.3
**Twanoh**	0.2 ± 49.2	−260.8 ± 118.1	−17.0 ± 107.0	46.4 ± 95.0	11.8 ± 85.8	40.2 ± 90.7	8.6 ± 78.3	14.8 ± 104.3
**Châ bá**	−1.7 ± 24.9	−7.8 ± 63.7	−53.3 ± 67.3	−12.2 ± 79.9	−55.8 ± 95.3	−17.7 ± 50.0	−55.9 ± 69.5	−46.6 ± 111.9
**M2**	−6.2 ± 23.8	−15.3 ± 33.6	−35.1 ± 60.6	−52.7 ± 72.2	−39.4 ± 48.0	−34.0 ± 43.4	−53.9 ± 48.7	−45.6 ± 69.7
**Kodiak**	2.7 ± 38.3	25.7 ± 83.6	14.1 ± 80.8	57.4 ± 111.7	24.5 ± 126.1	−1.6 ± 91.3	22.8 ± 89.4	47.4 ± 110.4
**GAKOA**	0.0 ± 9.9	0.1 ± 21.6	−67.3 ± 40.7	−63.3 ± 100.3	−71.2 ± 66.6	−10.5 ± 27.1	−63.8 ± 48.5	−53.3 ± 54.4
**Average**	**0.1 ± 23.6**	**−13.0 ± 49.2**	**−11.1 ± 58.8**	**4.6 ± 69.6**	**−2.9 ± 67.7**	**−2.2 ± 48.7**	**−7.2 ± 58.6**	**5.5 ± 72.1**

Only buoys within LMEs and with observations in more than 36 months of the timeseries were included. Grid cells nearest in space to the moored buoy coordinates were selected from each data product. If the nearest grid cell did not contain *p*CO_2_ values, the next nearest grid cell was selected. This process was repeated until the chosen grid cell contained *p*CO_2_ values.

**Fig. 12:**
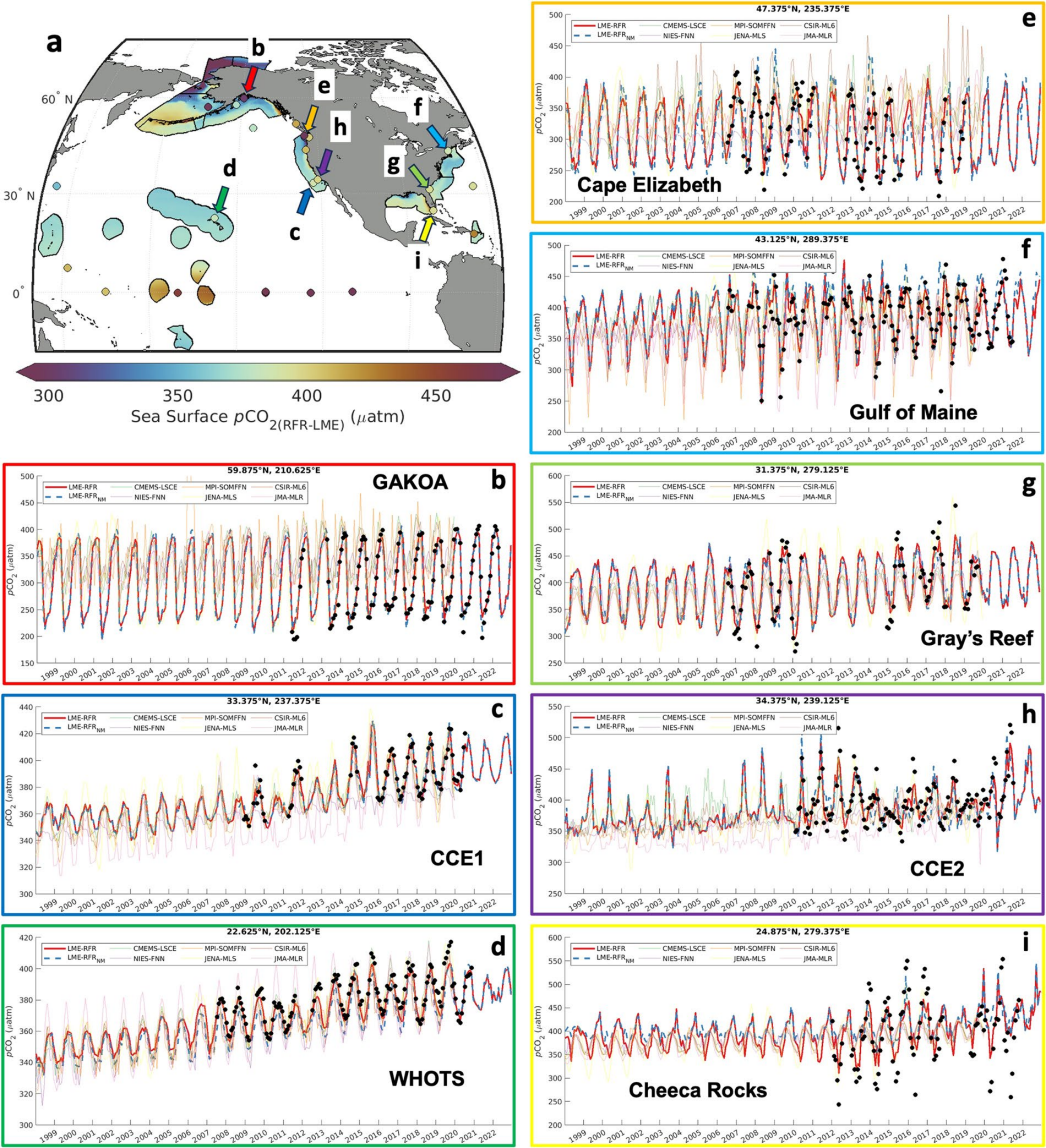
Comparisons between *p*CO_2_ from selected moored buoy observations, RFR-LME and RFR-LME-NM maps, and other mapped surface products. (**a**) Mapped long-term mean *p*CO_2(RFR-LME)_ along with mean *p*CO_2_ from moored buoy observations (shaded dots). Colors of the arrows in the map correspond to the colors of the outlines of timeseries plots from grid cells that match the buoy locations. (**b**–**i**) Each timeseries shows buoy observations aggregated into monthly bins (black dots), corresponding timeseries from RFR-LME maps (red solid lines) and RFR-LME-NM maps (blue dashed lines), and corresponding timeseries from mapped global data products included in SeaFlux^47^ (thin colored lines).

Individual timeseries from moored buoy sites (Fig. 12) emphasize the significant seasonal and interannual variability in buoy *p*CO_2_ observations (black dots), even when aggregated in monthly bins, and the challenge for mapped products (colored lines) to accurately capture each of those variations at a local scale. The performance of the regional RFR-LME maps compared to the global mapped products reinforces the notion that locally specific relationships captured by training machine learning algorithms at the scale of objectively defined clusters within LMEs can resolve fine-scale variations in ocean biogeochemistry more effectively than global-scale algorithms, even though those global-scale algorithms are trained with a larger amount of data^24,67^. Positive trends in *p*CO_2_ superimposed upon seasonal variations are visible in both the moored buoy observations and mapped data product timeseries (Fig. 12).

### Comparison to mapped data products

**Fig. 13:**
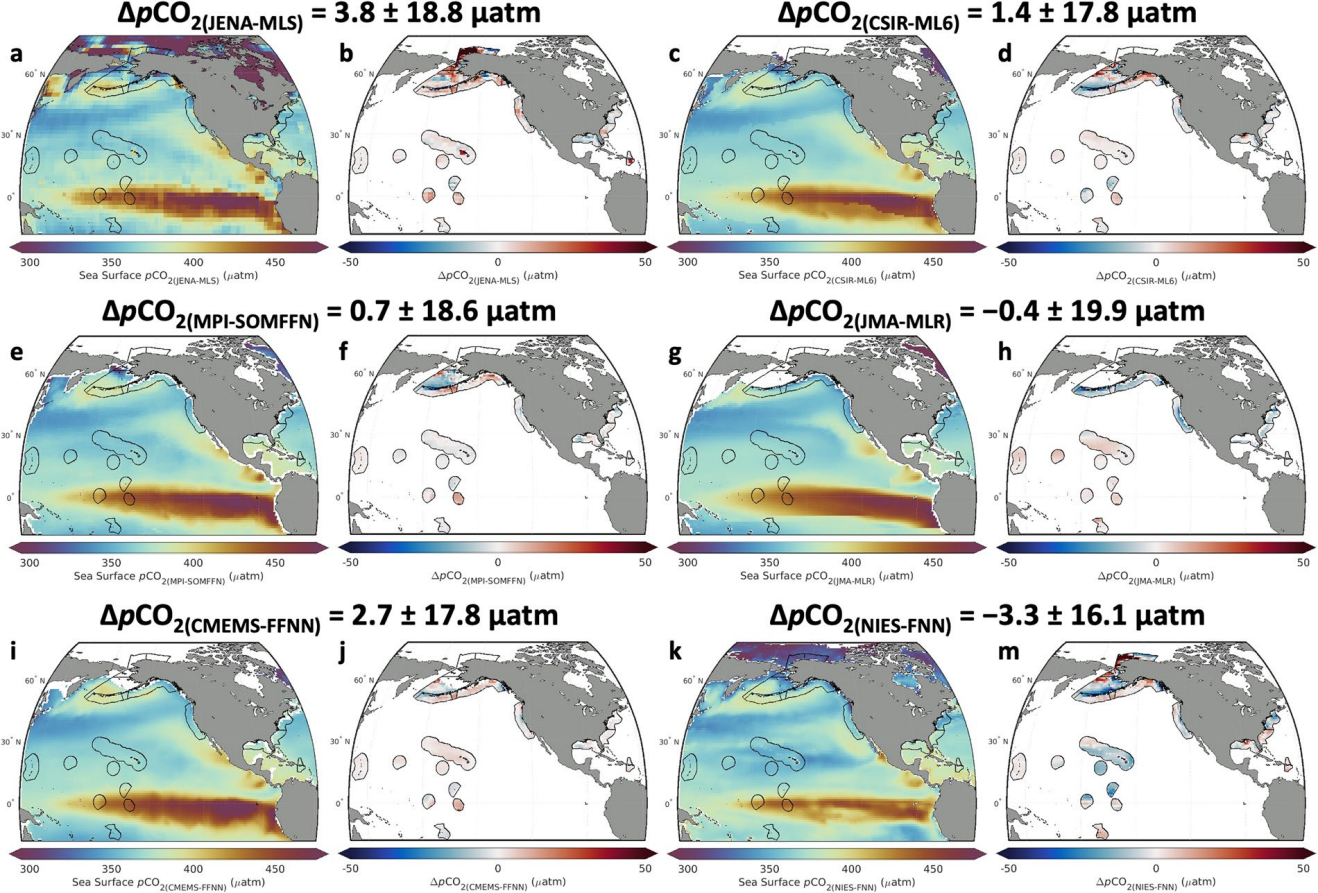
Comparisons between mapped surface *p*CO_2_ products and RFR-LME maps. Long-term mean *p*CO_2_ for each of the SeaFlux^47^ mapped global products (**a,c,e,g,i,k**) and differences between those products and RFR-LME (Δ*p*CO_2_ = *p*CO_2(SeaFlux)_ − *p*CO_2(RFR-LME)_; **b,d,f,h,j,m**) are shown. Area-weighted averages and standard deviations of Δ*p*CO_2_ are provided above each set of two figures.

Finally, RFR-LME surface *p*CO_2_ was compared directly to the six global-scale mapped products of *p*CO_2_ from SeaFlux across the overlapping interval between them (1998–2019). Maps of average surface *p*CO_2_ display similar patterns across all six SeaFlux products, but differences between those products and RFR-LME (Δ*p*CO_2_ = *p*CO_2(SeaFlux)_ − *p*CO_2(RFR-LME)_) reveal subtle regional differences (Fig. 13). SeaFlux provides a *p*CO_2_ filler field derived from Landschützer *et al*.^68^ to fill spatial gaps in global surface products; this gap filler is not used to produce the difference maps displayed in Fig. 13. However, for spatial consistency, it is used to calculate the averages and standard deviations of the differences for each data product shown in Fig. 13.

In the tropical Pacific, RFR-LME maps agreed well with all products but NIES-FNN, where a prevailing negative bias is evident in that product. In the Atlantic, RFR-LME maps generally agreed well, with visible biases in the Mississippi plume (CSIR-ML6), Georges Bank (JMA-MLR), Caribbean (JENA-MLS), and throughout (NIES-FNN). Coastal negative biases are visible for most products in the central CCS region, and coastal positive biases are visible in the northern CCS region. Both positive and negative biases occur in the regions surrounding Alaska, where low observational density likely leads to significant diversity in *p*CO_2_ estimates among the gap-filling approaches.

Despite these regional discrepancies with some individual products, the median (±1 IQR) Δ*p*CO_2_ for the ensemble average of all six SeaFlux products is 0.8 ± 16.6 μatm. This indicates that RFR-LME — which represents local-scale temporal variability in surface *p*CO_2_ more effectively than global products (Table 9; Fig. 11) — agrees at broad scales with observation-based products that are well accepted and widely used by community-wide synthesis efforts such at the Global Carbon Budget^2^ and REgional Carbon Cycle Assessment and Processes Project (RECCAP2)^69^.

## Data Availability

Code for accessing and processing the data discussed in this study is freely available on Github (https://github.com/jonathansharp/US-RFR-LMEs). Code was written in MATLAB version R2022a. Parameters used to generate and validate the current dataset are described throughout the Methods section and are listed in Table 2.
